# How Toll-like Receptor 9 Plays a Key Role in the Development of Gastric Cancer and Is Linked to Epstein–Barr Virus Infection

**DOI:** 10.3390/cancers15205104

**Published:** 2023-10-23

**Authors:** Marek Majewski, Paulina Mertowska, Sebastian Mertowski, Kamil Torres, Ewelina Grywalska

**Affiliations:** 1 Department of Plastic and Reconstructive Surgery and Microsurgery, Medical University of Lublin, 20-059 Lublin, Poland; 2 Department of Experimental Immunology, Medical University of Lublin, 20-093 Lublin, Poland; sebastian.mertowski@umlub.pl (S.M.);

**Keywords:** TLR-9 expression, gastric cancer, immune cell subsets, EBV infection, immune responses, histological grade, TNM stage, soluble TLR-9, prognostic implications

## Abstract

**Simple Summary:**

TLR-9, a vital component of our immune system, is found to be significantly involved in the progression of gastric cancer. This research also investigates its connection to EBV infection. By analyzing specific T and B immune cell groups that express TLR-9, along with the levels of soluble TLR-9 in the blood, the study compares these factors between individuals with gastric cancer and healthy volunteers. The aim is to understand how these immune markers relate to the severity of the cancer, its stage, type, and survival rates. Furthermore, the study delves into the presence of EBV genetic material and its potential impact on these immune responses. This research sheds light on the interplay between TLR-9, immune cell populations, and EBV in gastric cancer development.

**Abstract:**

The relationship between Toll-like receptor 9 (TLR-9) signaling and its involvement with Epstein–Barr virus (EBV) in gastric cancer (GC) is complex and currently under study. This research intended to understand TLR-9’s role in certain T and B lymphocytes and the serum levels of TLR-9 in GC patients versus healthy subjects. The team explored links between these immune markers and various GC traits, such as histological grade, tumor progression stages, cancer types, and survival rates. Additionally, the research sought to find if EBV genetic material influences these immune reactions. Using flow cytometry, TLR-9 levels in different immune cells were analyzed. At the same time, the amount of TLR-9 in the serum was determined. The results showed GC patients had varied TLR-9 levels compared to healthy subjects, with specific cells showing noticeable changes. When grouped by GC attributes, key relationships emerged between TLR-9 amounts, the histological grade, progression stages, and cancer types. A notable finding was the connection between TLR-9 levels and EBV genetic presence, suggesting possible interactions between TLR-9 responses and EBV-related GC processes. Survival data also hinted at TLR-9’s potential as a predictor linked to clinical traits. Overall, this research emphasizes TLR-9’s complex role in GC’s immune responses, pinpointing its interactions with particular cells, clinical features, and EBV. The study unveils a complex web affecting GC and paves the way for new treatment avenues targeting TLR-9 pathways.

## 1. Introduction

Gastric cancer (GC) remains a formidable global health challenge, with diverse etiological factors contributing to its complex pathogenesis [1,2,3]. Epidemiological data indicate that in 2020, in the United States alone, 127,211 people were diagnosed with stomach cancer, and the rate of new cases was 6.9 per 100,000 men and women per year. Moreover, the death rate was 2.8 per 100,000 men and women per year [4]. In 2020, Poland was in third place among Central and Eastern European countries in terms of the incidence of stomach cancer, just behind Ukraine and Russia. Data from the National Cancer Registry from 2019 indicate that 5.1 thousand people suffer from stomach cancer in Poland. In terms of incidence, gastric cancer ranks seventh in men and outside the top ten in women among all diagnosed malignancies. In terms of mortality, they rank fourth in men and seventh in women. Although there is currently a systematic decrease in the number of new cases and deaths due to this cancer, the incidence and mortality rate of stomach cancer in Poland are among the highest in Europe [5]. The causes of stomach cancer are unknown and are subject to many intensive interdisciplinary studies. A key factor in understanding cancer biology is the complex interaction between the immune system and the tumor microenvironment [6,7,8]. Toll-like receptors (TLRs), as sentinel components of the innate immune system, have garnered increasing attention due to their capacity to discern pathogen-associated molecular patterns (PAMPs) and initiate immune responses [9,10,11]. In particular, Toll-like receptor 9 (TLR-9) has emerged as a focal point in the crossroads of immunomodulation and cancer development [12,13]. TLR-9 is an endosomal receptor classically known for its recognition of CpG (cytosine–guanosine dinucleotide) motifs, which are considered PAMP patterns due to their abundance in microbial and viral genomes but their rarity in vertebrate genomes. Additionally, in their unmethylated form, they act as immunostimulants [14,15,16]. When activated, a complex process is set in motion that results in the production of cytokines, chemokines, and other immune mediators. These substances coordinate the body’s innate and adaptive immune responses. In the case of cancer, particularly gastric cancer, the role that TLR-9 plays in immunosurveillance and shaping the tumor microenvironment suggests that it may be a promising target for therapy and a useful prognostic marker [17,18].

Notably, the relationship between TLR-9 and GC takes on additional complexity upon Epstein–Barr virus (EBV) infection, recognized as a predisposing factor in certain gastric malignancies [19,20]. Gastric cancer and nasopharyngeal cancer are the only two epithelial cancers known to be associated with EBV infection. However, in contrast to the almost universal occurrence of nasopharyngeal cancer, only 8–10 percent of gastric cancers are EBV-positive [21,22]. The convergence of TLR-9 signaling and EBV presence introduces intriguing prospects of crosstalk between viral infection and immune activation. However, the precise mechanistic nuances underlying this interplay and their implications for GC progression and patient outcomes remain to be elucidated [23,24].

This study embarks on a comprehensive investigation into the multifaceted role of TLR-9 in GC immunopathogenesis, encompassing the dynamic expression patterns of TLR-9 among distinct T and B lymphocyte subsets. Additionally, we explore the concentrations of soluble TLR-9 within the serum milieu, providing a broader insight into systemic immune activation. By carefully analyzing immune parameters alongside clinical variables such as histological grade and TNM stage, we aim to uncover the complex connections between TLR-9 expression, disease severity, and the potential impact of EBV infection.

The findings of this study hold promise for enhancing our comprehension of GC progression at the nexus of immune signaling and viral co-factors. Ultimately, a more nuanced understanding of TLR-9’s role in the GC microenvironment could provide novel avenues for therapeutic interventions and prognostic assessments, fostering a more targeted approach to managing this complex malignancy.

## 2. Materials and Methods

### 2.1. Patient Characteristics

The study group comprised 40 people diagnosed with GC, including 16 women and 24 men. The mean age of the patients in the study group was 62.10 ± 10.52 (median: 63, range 40–81). The type of cancer, stage based on the TNM scale, and grade were also specified in detail. The control group consisted of 30 healthy volunteers, including 13 women and 17 men. The mean age of healthy volunteers was 63.87 ± 9.56 (median: 64, range: 45–80). Patients with GC were recruited by a gastroenterologist based on the adopted guidelines published by the European Society for Medical Oncology (ESMO) and based on the guidelines and recommendations of Polish specialists “Polish consensus on the diagnosis and treatment of gastric cancer—update 2022” [25]. Patients from the control group were healthy volunteers without gastrointestinal or immune system diseases who, after giving written consent and undergoing examination by the referring physician, were included in this study. All patients included in the study were subject to a detailed assessment by the attending physician based on specific inclusion and exclusion criteria.

To ensure accurate results for both the patient and healthy volunteer groups, we implemented strict exclusion criteria. Individuals who had used medications that affect the immune system, undergone hormonal therapy, experienced an infection within the three months before the study, received prior blood transfusions, had autoimmune diseases, cancer, allergies, or had been pregnant or lactating within one year before the study were excluded from participation. This approach was taken to maintain the integrity of the study and ensure reliable outcomes.

The research material used in these analyses was peripheral blood collected into tubes with EDTA anticoagulant (10 mL) (used for immunophenotyping analyses and the isolation of peripheral blood mononuclear cells (PBMCs) and into clot tubes (5 mL)) in order to obtain serum. PBMCs were used to isolate genetic material in the form of DNA, which was used to assess the number of EBV virus copies, while serum was used to assess the concentration of the soluble form of TLR-9. Every participant willingly provided their informed consent for the study, which was conducted in line with the standards set by the Declaration of Helsinki and was sanctioned by the Ethics Committee of the Medical University of Lublin, Poland (approval no. KE-0254/251/2014). To ensure the privacy of the individuals involved, all patient samples were labeled using the laboratory’s specialized numbering system.

### 2.2. Immunophenotyping of TLR-9

**Figure 1:**
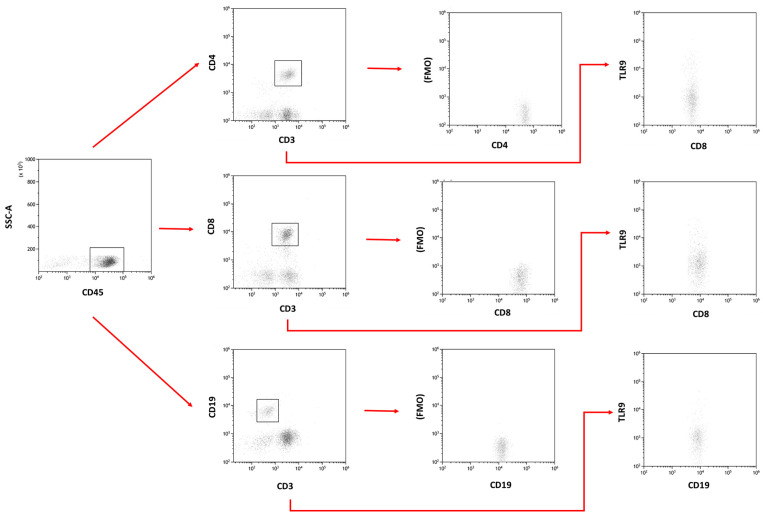
Example gating strategy and percentage of CD4+/CD3+, CD8+/CD3+, and CD19+/CD3− cells positive for expression of the TLR-9 molecule.

Flow cytometry evaluated the percentage of TLR-9-positive CD4+/CD8+/CD19+ lymphocytes in a peripheral blood sample. The whole blood sample was incubated with human monoclonal antibodies. These antibodies included anti-CD45 AF700, anti-CD4 BV421, anti-CD8 BV605, anti-CD19 FITC, anti-CD56 BV650, and anti-CD16 BV650, as well as anti-CD3 PerCp and anti-TLR-9 APC (BioLegend, San Diego, CA, USA). After the incubation stage, the samples were treated with a previously prepared lysing solution to remove red blood cells, and then the samples were washed twice. The thus prepared samples were evaluated with the CytoFLEX LX instrument (Beckman Coulter, Indianapolis, IN, USA). The obtained data were analyzed using the Kaluza Analysis program. An exemplary analysis can be found in Figure 1.

### 2.3. Isolation of PBMCs and Assessment of EBV Copy Number

The EBV DNA copy number was determined utilizing the ISEX variant of the EBV PCR kit manufactured by GeneProof, Czech Republic. Each sample was assessed twice, and a negative control consisting of DNA elution buffer was included. The specific conserved DNA sequence for the EBV nuclear antigen 1 gene (EBNA1) was amplified utilizing the 7300 Real-Time PCR System produced by Applied Biosystems, USA, in accordance with the instructions provided with the ISEX EBV variant PCR kit. The viral DNA copy number per microliter of the eluent was adjusted to account for DNA isolation efficiency and expressed as viral DNA copy number per microgram of DNA obtained. Samples below the detection threshold of ten EBV DNA copies per microliter were classified as negative for EBV.

### 2.4. Evaluation of sTLR-9 Concentration

To determine the serum concentration of the soluble form of TLR-9 in patients enrolled in the study, an enzyme immunoassay was performed using the commercially available Human Toll-Like Receptor 9 (TLR-9) ELISA Kit (MyBioSoure, San Diego, CA, USA, Cat No. MBS268453). Detection range: 20 ng/mL–0.312 ng/mL; sensitivity: up to 0.06 ng/mL. The assay was performed according to the manufacturer’s instructions using the ELISA VICTOR microplate reader.

### 2.5. Statistical Analysis

The study’s data were analyzed using Tibco Statistica 13.3 software (Palo Alto, CA, USA). The distribution of the data was assessed using the Shapiro–Wilk test. The Kruskal–Wallis test was employed to compare the groups, followed by Dunn’s post hoc test. Dunn’s test *p*-values were adjusted for multiple comparisons using the Bonferroni method. To explore the relationships between variable pairs, Spearman’s correlation coefficients were calculated. ROC curves were used to assess the laboratory test’s diagnostic performance for patient-related parameters. The analysis of the influence of the studied parameters on patient survival was performed using the Kaplan–Meier method in the GraphPad Prism software, version 9.4.1 (San Diego, CA, USA).

## 3. Results

### 3.1. Characteristics of GC Patients and Healthy Volunteers Enrolled in the Study

**Figure 2:**
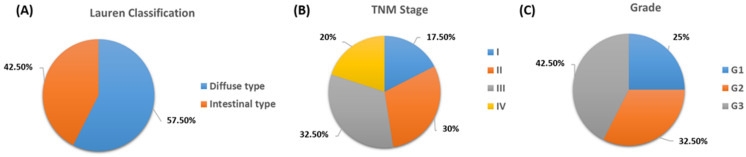
Detailed analysis of selected parameters of GC patients. (**A**) Lauren Classification, (**B**) TNM stage, (**C**) Grade.

All patients included in the analysis were subject to the detailed inclusion and exclusion criteria described in Section 2. The study group consisted of 40 newly diagnosed GC patients aged 40 to 81 with a median of 63 years. GC staging included grade, stage based on the TNM scale and Lauren classification. Gastric cancer can be divided into different histological types based on the characteristics of the tumor cells and the growth pattern based on the Lauren classification [26]. The two main histological types of GC are the diffuse type and the intestinal type, and their identification has important implications for tumor behavior, prognosis, and treatment strategy selection. Diffuse GC is characterized by solitary tumor cells that infiltrate the stomach wall without forming distinct masses or glands. Cancer cells often have a signet ring appearance, where the nucleus is pushed to the periphery of the cell by droplets of mucin, giving the cell a “signet ring” appearance under the microscope. This type of GC tends to be more aggressive and may be more likely to spread to nearby tissues and organs [27]. Diffuse gastric cancer is often associated with genetic mutations and molecular changes, including mutations in the *CDH1* gene, which is associated with hereditary diffuse GC [28]. Intestinal-type GC is characterized by the formation of distinct glandular structures that resemble the normal lining of the stomach. It often develops in areas of chronic gastritis or intestinal metaplasia, which are precancerous conditions. Intestinal-type GC tends to grow more slowly and is often associated with risk factors such as *Helicobacter pylori* infection and dietary factors. This type of GC can sometimes be detected at an earlier stage, which can improve treatment outcomes [29]. In our study, more than 57.50% of recruited patients had the diffuse type at diagnosis, and the remaining 42.50% had the intestinal type (Figure 2A). The mean age of patients with diffuse type GC was 61.70 ± 9.71 (median: 63, range: 40–77) and intestinal type 64.06 ± 11.38 (median: 66, range: 41–81).

Another aspect was the assessment of the severity of GC based on the TNM scale. The TNM staging system is a widely used system to describe the spread of cancer in a patient’s body and provides important information to help guide treatment decisions and provide insight into prognosis [30]. Analysis of the detailed data of GC patients showed that 17.5% of the patients were in TNM I (median age: 67, range: 49–76); 30% in TNM II (median age: 62.5, range: 41–81), 32.5% in TNM III (median age: 64, range: 75–76) and 20% in TNM IV (median age: 60, range: 40–77) (Figure 2B). In addition, the grade of recruited patients was determined, which was 25% of patients for G1 (median age: 69, range 41–81); for G2 32.5% of patients (median age: 65, range: 43–76) and for G3 42.5% of patients (median age: 62, range: 40–77) (Figure 2C).

The control group consisted of 30 healthy volunteers matched in terms of age to the study group. The next stage of our analyses was the evaluation of selected peripheral blood parameters of both populations, with particular emphasis on the assessment of the immunophenotype. The obtained results were collected and are presented in Table 1.

We showed statistically significant differences between the studied populations for the value of white blood cells and the percentage of NK cells CD3-CD16+CD56+ and T lymphocytes CD3+CD4+. A detailed analysis of GC patients, taking into account Lauren’s classification (Table 2), grade (Table 3) and TNM stage (Table 4), also showed a number of significant changes in the study population.

In the first case, we observed statistically significant changes for the level of white blood cells and monocytes, as well as the T lymphocyte ratio CD3+CD4+/T CD3+CD8+, which were higher in patients with diffuse type than in intestinal type. The opposite trend was observed for the percentage of NK cells CD3-CD16+CD56+, NKT-like cells CD3+CD16+CD56+ and T lymphocytes CD3+CD8+ (Table 2).

The second analyzed parameter, i.e., grade, showed in most of the analyzed parameters a statistically significant increase in white blood cells and lymphocytes and the percentage of CD3+CD4+ T lymphocytes, as well as the T lymphocyte ratio CD3+CD4+/T CD3+CD8+ with an increase in grade. A statistically significant decrease in the value with increasing grade was recorded for the percentage of NK cells CD3-CD16+CD56+, NKT-like cells CD3+CD16+CD56+ and T lymphocytes CD3+CD8+ (Table 3). Similar trends were also maintained for the TNM stage analysis (Table 4).

### 3.2. Analysis of the Percentage of TLR-9 on Selected Subpopulations of T and B Lymphocytes in GC Patients in Relation to Healthy Volunteers

Next, we analyzed the occurrence of CD4+ and TCD8+ T cells and CD19+ B cells positive for TLR-9 expression. The primary analysis between GC patients and healthy volunteers is presented in Table 5.

**Figure 3:**
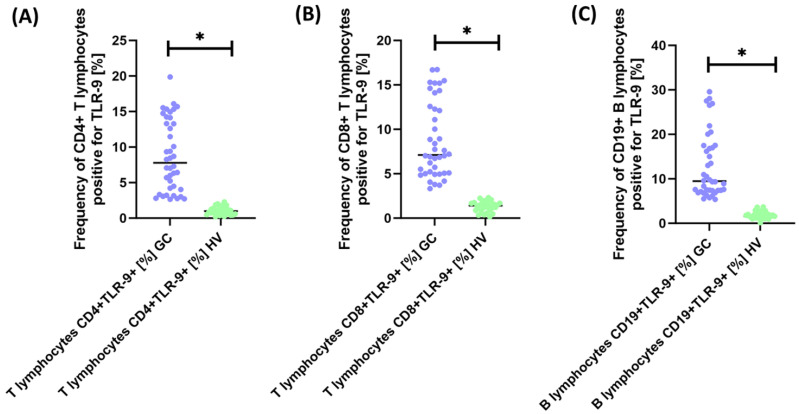
Analysis of the percentage of TLR-9 on selected subpopulations of T (**A**,**B**) and B (**C**) lymphocytes in GC patients relative to healthy volunteers. Blue is for GC patients and green is for healthy volunteers. Statically significant results are marked *.

As we can see, all analyzed lymphocyte subpopulations showed a significant increase in TLR-9 expression of 7.86-fold for CD4+TLR-9+ T lymphocytes (Figure 3A), 5.05-fold for CD8+TLR-9+ T lymphocytes (Figure 3B), and 5.81-fold for CD19+TLR-9+ B lymphocytes (Figure 3C).

Due to such significant changes, we decided to take a closer look at the changes in the examined parameter, taking into account the Lauren classification (Table 6), grade (Table 7) and TNM stage (Table 8).

The analysis of GC patients by diffuse type and intestinal type showed no statistically significant changes in the percentage of TLR-9-positive lymphocytes (Supplementary Materials Figure S1).

Analysis of the parameters tested in GC patients by grade showed a significant increase in T lymphocytes CD4+TLR-9+ and B lymphocytes CD19+TLR-9+ between G1 and G2 and G3 patients (Supplementary Materials Figure S2A–C). No statistically significant changes were noted for T lymphocytes CD8+TLR-9+ between each grade and for all tested parameters between G2 and G3.

The last analysis concerned the differences between GC patients by TNM stage. Our studies showed that for T lymphocytes CD4+TLR-9+ (Supplementary Materials Figure S3A) and B lymphocytes CD19+TLR-9+ (Supplementary Materials Figure S3C), there was a statistically significant increase in the values between TNM I and TNM III and TNM IV as well as TNM II and TNM III and TNM IV. For T lymphocytes CD8+TLR-9+, there was a significant increase in TNM I vs. TNM III and TNM II vs. TNM III as well as TNM II vs. TNM IV (Supplementary Materials Figure S3B).

### 3.3. Evaluation of the Concentration of the Soluble Form of sTLR-9 in Patients with GC in Relation to Healthy Volunteers

**Figure 4:**
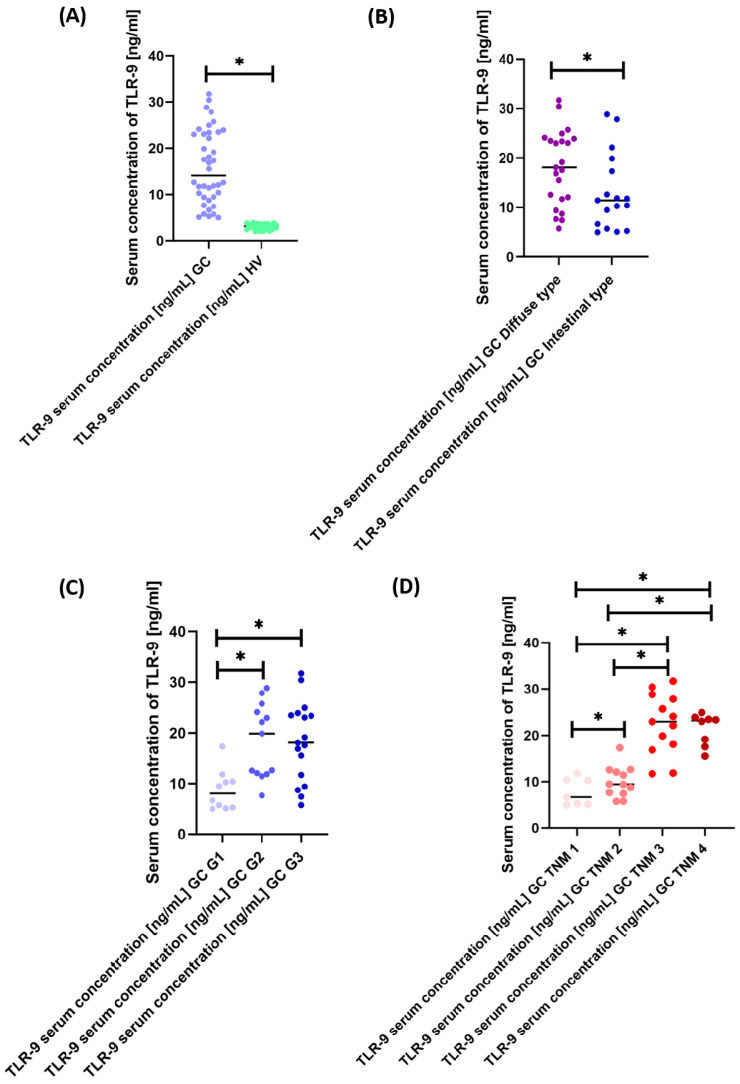
Graphical representation of TLR-9 serum concentration results in GC patients versus healthy volunteers (**A**) and by Lauren classification (**B**), grade (**C**) and TNM stage (**D**). For ease of interpretation, color markings are used, and statistically significant results are marked *.

**Figure 5:**
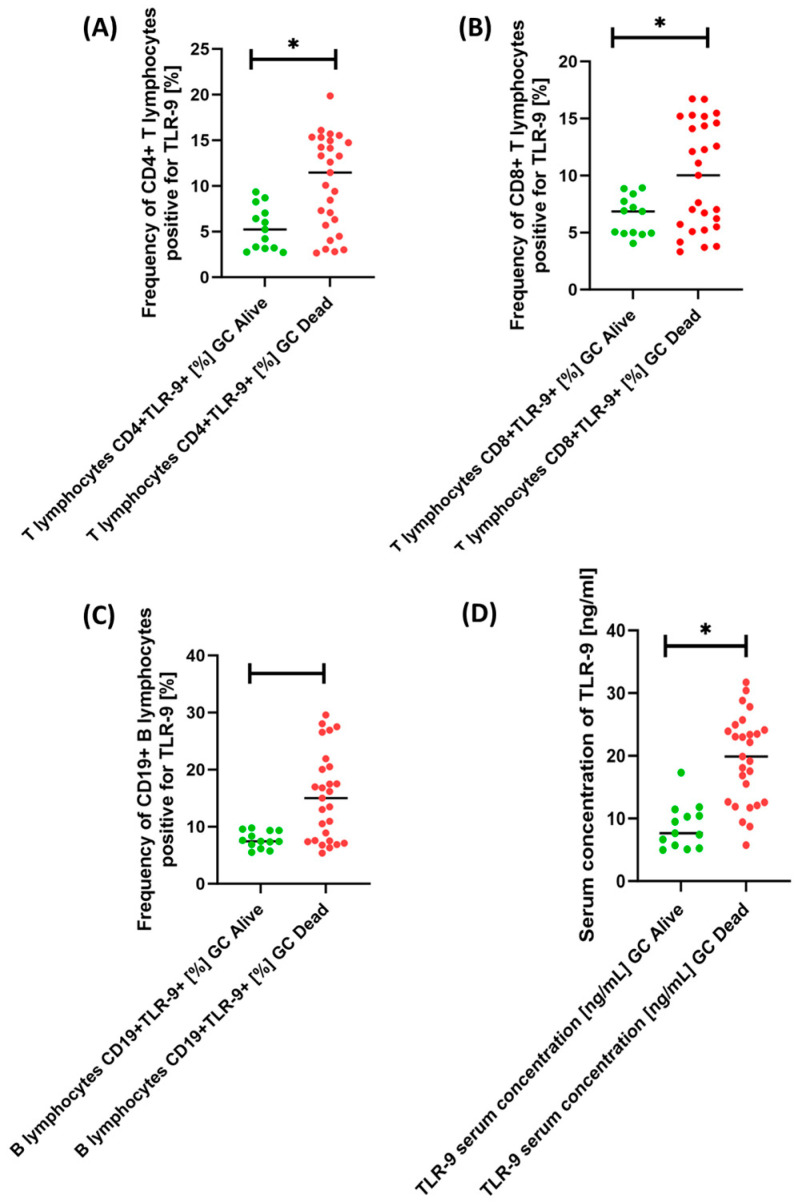
Percentage of T cells (**A**,**B**) and B cells (**C**) positive for TLR-9 expression and assessment of serum concentration (**D**) in GC patients by survival status. For better interpretation of the results, patients who did not survive are marked red and patients who survived are marked green. Statistically significant results are marked *.

Analysis of the concentration of the soluble form of TLR-9 between GC patients and healthy volunteers showed a significant increase in its concentration of 4.46-fold in GC patients (Figure 4A). Moreover, a detailed analysis of GC patients according to Lauren classification and grade and TNM stage also showed significant differences. In the first case, the concentration of sTLR-9 was 1.58-fold higher in diffuse-type patients compared to intestinal-type (Figure 4B). In the second case, the concentration of the tested molecule was the highest in G2 patients by 2.46 times compared to G1 and slightly higher than in G3 patients. The concentration difference between patients with G1 versus G3 was 2.24-fold (Figure 4C). In the last analyzed aspect, we observed that the concentration of sTLR-9 also increased with the increase in the TNM stage. These differences were as follows: 1.42 times higher for TNM II than for TNM I; 3.45 times higher for TNM III than for TNM I; 3.49 times higher for TNM IV compared to TNM I; 2.43 times higher between TNM III and TNM II; and 2.46-fold higher between TNM IV and TNM II (Figure 5D). Detailed research data are presented in Supplementary Materials Table S1.

### 3.4. Effect of EBV on TKLR-9 Related Parameters in GC Patients

Another aspect of the study was the assessment of the effect of EBV on the analyzed immunological parameters associated with TLR-9 in GC patients. Out of 40 patients included in the study, as many as 19 (47.5%) patients showed the presence of the number of EBV virus copies in the tested material. In patients in the control group, the absence of EBV was below the limit of detection of the test (i.e., fewer than 10 copies of the virus). Due to the presence of EBV in the genetic material of patients, we decided to take a closer look at the analyzed parameters by grouping GC patients into EBV+ and EBV−. The obtained results are presented in Table 9.

Based on the obtained results, we can see that patients with GC EBV+ are dominated by diffuse type, grade G3 and TNM III stage, in contrast to patients with GC EBV−, for whom intestinal type, grade G1 and TNM II stage are the most common. Statistically significant differences in peripheral blood analyses between these groups were observed for the level of white blood cells and the percentage of NK cells CD3-CD16+CD56+ and NKT-like cells CD3+CD16+CD56+ or T lymphocytes CD3+CD8+. The most significant differences concerned the percentage of tested lymphocytes showing positive expression of TLR-9. All tested lymphocyte subpopulations showed a higher positive expression of the tested TLR in GC EBV+ patients in relation to GC EBV−. These changes were as follows: 3.35 times higher for T lymphocytes CD4+TLR-9+; 2.47 times for T lymphocytes CD8+TLR-9+; and 2.37 times for B lymphocytes CD19+TLR-9+. In addition, the concentration of TLR-9 in the serum of GC EBV+ patients was 2.47 times higher than in GC EBV− patients.

### 3.5. Importance of TLR-9 as a Prognostic Marker

Based on the information obtained, we were able to determine whether the patients included in this study are still alive. It turned out that out of 40 patients included in the study, only 13 survived, i.e., 32.5%. Therefore, we decided to take a closer look at our results in terms of survival status. The obtained test results are presented in Table 10.

Based on the collected data, we can see that the patients who did not survive at the time of diagnosis had predominantly diffuse type, grade G2 and G3 and TNM III stage, while the patients who survived at the time of diagnosis were predominantly of intestinal type, grade G1 and TNM I and TNM II. In addition, all patients who died were included in the GC EBV+ patient group. Of particular note is the importance of TLR-9. As we can see, patients from the Dead GC group were characterized by an increased percentage of T and B lymphocyte subpopulations tested positive for TLR-9 expression compared to Alive GC patients. These changes were as follows: 2.19-fold for CD4+TLR-9+ T lymphocytes (Figure 5A); 1.46-fold for CD8+TLR-9+ T lymphocytes (Figure 5B); and 2.01-fold for B lymphocytes CD19+TLR-9+ (Figure 5C). Moreover, the serum concentration of the tested receptor was also 2.60-fold higher in the patients who died relative to the patients who survived (Figure 5D).

**Figure 6:**
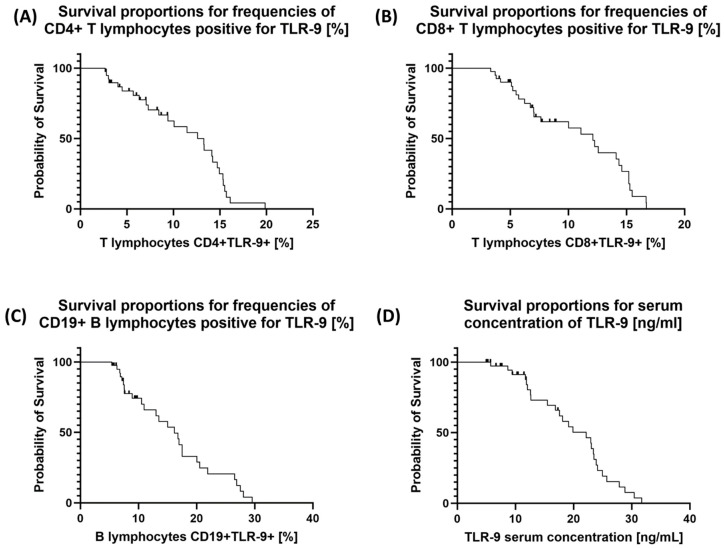
Probability of survival analysis for selected subpopulations of TLR-9-positive T cells (**A**,**B**) and B cells (**C**) and for serum TLR-9 concentration (**D**) in GC patients by their survival status.

To confirm our observations, we performed a Kaplan–Meyer analysis, which is illustrated in Figure 6. As assumed, the higher the percentage of TLR-9-positive T and B cells and the higher the concentration of TLR-9 in serum, the lower the probability of survival.

### 3.6. How Does TLR-9 Affect Clinical Parameters? A Correlation Assessment

In the further stages of the analysis, we decided to check whether the TLR-9 results obtained by us affect the clinical parameters of GC patients. As in the above cases, we also analyzed all parameters of Lauren classification, grade, TNM stage and patient survival status. Due to the amount of data received, details are provided in the Supplementary Materials.

Based on the obtained results, we can conclude that in the case of patients who did not survive, TLR-9 significantly correlated with Survival time from diagnosis, Stage TNM and EBV copy number (Supplementary Materials Table S3). Similar trends were observed for diffuse-type patients (Supplementary Materials Table S4), G2 and G3 (Supplementary Materials Tables S7 and S8) and TNM III and IV (Supplementary Materials Tables S11 and S12).

### 3.7. ROC Curve Analysis

**Figure 7:**
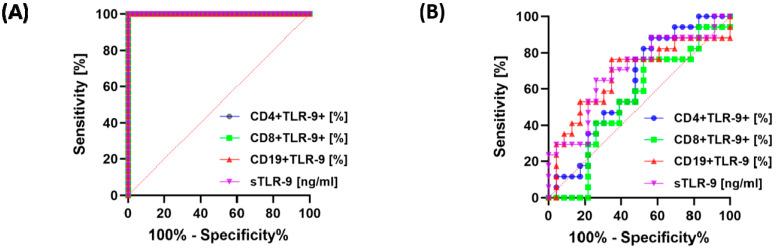
Analysis of ROC curves of frequencies of CD4+/CD8+ T lymphocytes and CD19+ B lymphocytes positive for TLR-9 and serum concentration of TLR-9 in GC patients versus healthy volunteers (**A**) between diffuse and intestinal GC types (**B**).

In the next stage of our analysis, we decided to check whether the expression of TLR-9 on selected subpopulations of immune cells and the serum concentration of the tested molecule can serve as a good diagnostic molecule for GC patients, with particular emphasis on type, grade, TNM stage and survival. All parameters analyzed showed high sensitivity and specificity in GC patients compared to healthy volunteers (Figure 7A). A detailed analysis of the immunological parameters in GC patients, taking into account the type of tumor, showed that none of them is a specific parameter allowing one to clearly distinguish GC diffuse type from GC intestinal type (Figure 7B).

**Figure 8:**
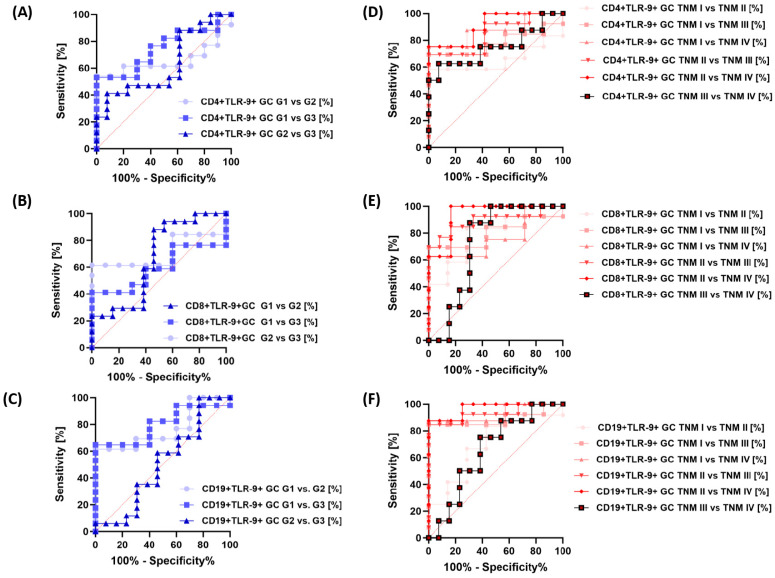
Analysis of ROC curves for the percentage of TLR-9-positive lymphocyte subpopulations in GC patients by grade and TNM stage. (**A**) Frequencies of CD4+ T lymphocytes positive for TLR-9 among GC G1-G3 patients, (**B**) Frequencies of CD8+ T lymphocytes positive for TLR-9 among GC G1-G3 patients, (**C**) Frequencies of CD19+ T lymphocytes positive for TLR-9 among GC G1-G3 patients, (**D**) Frequencies of CD4+ T lymphocytes positive for TLR-9 among GC TNM I–TNM IV, (**E**) Frequencies of CD8+ T lymphocytes positive for TLR-9 among GC TNM I–TNM IV, (**F)** Frequencies of CD19+ B lymphocytes positive for TLR-9 among GC TNM I–TNM IV.

**Figure 9:**
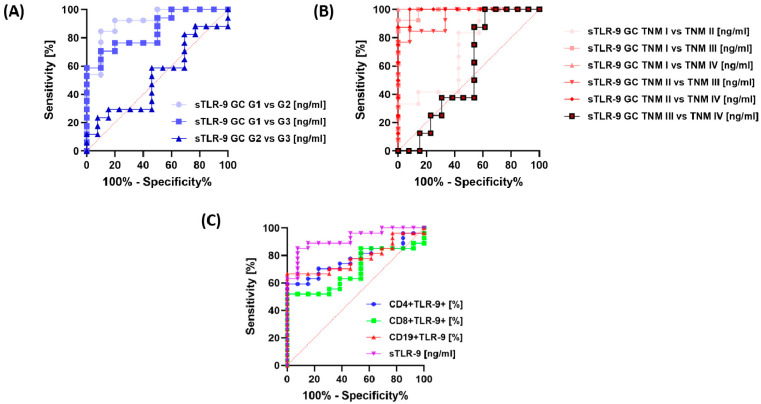
Analysis of ROC curves for TLR-9 serum concentration in GC patients by grade (**A**) and TNM stage (**B**), as well as analysis of the percentage of TLR-9-positive lymphocyte subpopulations and their serum concentration in GC patients, taking into account their survival (**C**).

Similar trends were observed for the analysis of GC patients by grade (Figure 8A–C) and TNM stage (Figure 8D–F), as well as for sTLR-9 serum concentration (Figure 9A,B). The analysis of patients in terms of their survival showed that the most sensitive of all analyzed parameters was the concentration of sTLR-9 in the serum of GC patients (Figure 9C).

**Figure 10:**
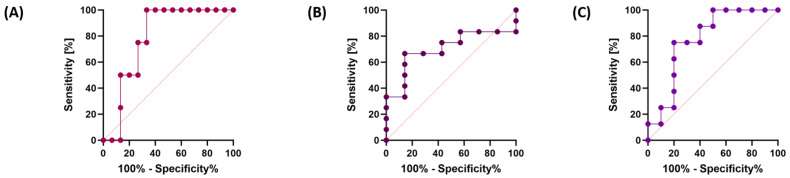
Analysis of ROC curves for EBV genetic material in GC patients by diffuse and intestinal type (**A**), G2 and G3 grade (**B**) and TNM 3 and TNM4 stage (**C**).

Due to the occurrence of EBV genetic material in selected GC patients, the analysis of the ROC curves for individual parameters such as type, grade and stage of TNM is presented in Figure 10. We can see that this EBV number is the most sensitive in the analysis of GC diffuse type and GC intestinal type compared to other parameters.

Such diversified results of ROC analyses may be due to the small number of patients in particular groups of analyzed parameters of GC patients, which underlines the importance of continuing research on this subject.

## 4. Discussion

It should be noted that the relationship between TLR-9, EBV, and gastric cancer is complex and not fully understood. Although TLR-9 activation can initiate an immune response against EBV, it may also contribute to chronic inflammation that may promote carcinogenesis. Additionally, the precise mechanisms by which EBV contributes to the development of gastric cancer are still an active area of research, and there may be individual differences in how TLR-9 responds to EBV infection.

In the presented studies, we observed differences in TLR-9 expression between T and B cell subsets, highlighting the heterogeneous nature of immune responses in the GC microenvironment [31,32,33]. This heterogeneity may reflect different stages of tumor-induced immune activation or immune suppression. In particular, significant correlations between TLR-9 expression levels and clinicopathological variables, such as histological grade and tumor–node–metastasis (TNM) stage, highlight the potential prognostic importance of TLR-9 in GC.

TLR-9 can recognize EBV DNA in endosomes when the virus infects and replicates in host cells. Activation of TLR-9 triggers an immune response, including the production of pro-inflammatory cytokines and type I interferons, which aim to fight the virus. However, chronic TLR-9 activation can lead to persistent inflammation, a known risk factor for cancer development, including GC. The identification of serum-soluble TLR-9 provides a systemic perspective on TLR-9-mediated immune responses. Increased levels of soluble TLR-9 in GC patients compared to healthy controls may indicate increased immune activation in the tumor microenvironment [18]. However, further research is warranted to elucidate the precise source and biological significance of soluble TLR-9, potentially increasing its utility as a diagnostic or prognostic biomarker.

EBV genetic material modulates TLR-9 expression patterns, suggesting a potential link between viral infection and immune system activation [15,20,34,35]. Although the mechanistic basis of this interaction remains elusive, it raises possibilities regarding the role of TLR-9 as a sensor of viral invasion and coordinator of immune responses against EBV-infected cells [36,37]. In cases where EBV persists in the gastric mucosa and continuously activates TLR-9, the sustained inflammatory response may contribute to transforming normal gastric epithelial cells into cancer cells. The continued production of inflammatory mediators can damage DNA and promote cell proliferation and survival, all of which are hallmarks of cancer development.

Looking ahead, the intricate interplay between study molecules and EBV in immunopathogenesis of gastric cancer opens up a realm of exciting avenues for further research and translational applications. First of all, delving deeper into the molecular mechanisms underlying TLR-9-mediated immune responses and its connection to EBV infection represents a pivotal future endeavor. By delving into the complex signaling networks and possible viral manipulation of TLR-9 pathways, new targets for therapeutic interventions could be discovered. With the existing connections between TLR-9 expression, clinicopathological variables, and the presence of EBV, the development of targeted therapies becomes more promising. Designing interventions that harness TLR-9 activation to enhance immune responses against EBV-infected cancer cells could revolutionize GC treatment strategies. Moreover, the complex interplay between TLR-9, immune cell subsets, and EBV infection underscores the need for personalized approaches in GC management. Integrating TLR-9 profiling with other molecular markers could enable tailored treatments that capitalize on individual immune dynamics and tumor characteristics. Expanding the exploration of TLR-9’s prognostic value could refine risk stratification and guide clinical decision-making. Correlating TLR-9 expression with long-term survival outcomes and treatment responses could lead to more accurate prognostic tools. It is hoped that long-term studies tracking TLR-9 expression and EBV presence over time may uncover dynamic changes in immune responses during disease progression or in response to therapies. Such insights may aid in adaptive treatment strategies and shed light on the evolving immune landscape. Identifying robust biomarkers associated with TLR-9 expression and EBV presence could facilitate non-invasive diagnostics and monitoring. Blood-based assays targeting soluble TLR-9 or immune cell profiles could offer valuable tools for early detection and disease monitoring.

## 5. Conclusions

Investigating the complex relationship between TLR-9, immune cells, and EBV is essential in the study of GC immunopathogenesis. Through a comprehensive exploration of TLR-9 expression patterns across distinct T and B lymphocyte subsets, coupled with the quantification of soluble TLR-9 in serum, this study illuminated the multifaceted role of TLR-9 in shaping immune responses within the context of GC. The correlations established between TLR-9 expression and clinicopathological variables, including histological grade, tumor–node–metastasis (TNM) stage, and EBV presence, underscore the complexity of TLR-9’s engagement in GC development.

The nuanced interactions observed between TLR-9, immune cells, and viral co-factors warrant further investigation into the underlying mechanisms governing immune activation and potential viral modulation. The tantalizing prospect of deciphering the precise crosstalk between TLR-9-mediated signaling and EBV infection holds the potential to unravel new avenues for therapeutic strategies tailored to the unique molecular milieu of GC.

However, although our study expands the knowledge of TLR-9 in the context of GC, it has several limitations. The study’s cross-sectional nature limits the possibility of concluding that there is a causal relationship between TLR-9 expression, clinicopathological features, and the presence of the EBV virus. First of all, it would be necessary to repeat these studies by increasing the number of patients with GC, enabling the assessment of the observed relationships in a larger population and drawing much more precise conclusions. Furthermore, steps should be taken to monitor study patients and changes in the immune system over a longer time frame, taking into account the impact of treatment or surgical interventions and monitoring the number of infections. Due to limitations, these studies are preliminary studies signaling specific mechanisms occurring during the immunopathogenesis of GC.

In conclusion, advances in cancer immunology require understanding the complex connections between immune signaling, viral infections, and tumor progression. This study’s findings contribute to this expanding knowledge base, shedding light on the role of TLR-9 as a dynamic modulator of immune responses in the context of GC. Ultimately, the insights gleaned from this investigation could pave the way for targeted interventions, prognostic assessments, and personalized treatments aimed at altering the trajectory of GC, thereby addressing a critical unmet need in the realm of oncology.

Essentially, these studies provide the basis for a multidisciplinary exploration of TLR-9 convergence, immune responses, and viral infection in GC. We sincerely hope that these insights will also encourage other scientists to explore uncharted territories, develop innovative therapeutic strategies, and contribute to the growing knowledge aimed at changing the management of GC. The interplay between TLR-9, immune dynamics, and EBV infection could revolutionize GC pathogenesis and clinical management in the near future.

## Figures and Tables

**Table 1:** Evaluation of selected parameters of peripheral blood of patients with GC in relation to healthy volunteers.

Parameters		GC Patients (*n* = 40)		HV Patients (*n* = 30)		*p*-Value
		Mean ± SD	Median (Range)	Mean ± SD	Median (Range)	
Morphology	White blood cells [10^3^/mm^3^]	7.82 ± 1.22	7.64 (5.99–10.20)	7.17 ± 0.60	7.17 (5.40–8.49)	0.040 *
	Monocytes [10^3^/mm^3^]	0.48 ± 0.12	0.48 (0.22–0.70)	0.39 ± 0.13	0.48 (0.10–0.75)	0.901
	Lymphocytes [10^3^/mm^3^]	2.25 ± 0.47	2.23 (1.47–3.14)	2.44 ± 0.59	2.54 (1.46–4.10)	0.195
Immunophenotype	T lymphocytes CD3+ [%]	72.23 ± 3.09	72.82 (66.16–79.79)	72.03 ± 2.84	72.09 (65.34–77.51)	0.809
	B lymphocytes CD19+ [%]	11.16 ± 2.36	10.98 (8.21–16.30)	12.12 ± 2.18	12.24 (7.62–16.82)	0.082
	NK cells CD3-CD16+CD56+ [%]	11.76 ± 2.70	12.03 (6.11–17.24)	13.50 ± 3.48	13.72 (7.13–19.65)	0.019 *
	NKT-like cells CD3+CD16+CD56+ [%]	2.70 ± 1.765	2.19 (0.57–6.55)	3.17 ± 0.93	3.25 (1.29–4.95)	0.064
	T lymphocytes CD3+CD4+ [%]	39.64 ± 3.01	39.62 (34.53–47.08)	42.43 ± 5.78	41.69 (34.97–64.31)	0.025 *
	T lymphocytes CD3+CD8+ [%]	28.90 ± 3.95	29.17 (22.28–36.44)	29.62 ± 3.38	30.04 (22.25–35.78)	0.439
	T lymphocytes ratio CD3+CD4+/T CD3+CD8+	1.41 ± 0.27	1.36 (1.02–2.02)	1.46 ± 0.32	1.37 (1.03–2.13)	0.649

* Statistically significant results.

**Table 2:** Comparison of selected peripheral blood parameters of patients with GC, taking into account the Lauren classification.

Parameters		Diffuse-Type GC Patients (*n* = 23)		Intestinal-Type G Patients (*n* = 17)		*p*-Value
		Mean ± SD	Median (Range)	Mean ± SD	Median (Range)	
Morphology	White blood cells [10^3^/mm^3^]	8.30 ± 1.21	8.48 (5.99–10.20)	7.17 ± 0.89	7.39 (6.06–9.57)	0.008 *
	Monocytes [10^3^/mm^3^]	0.51 ± 0.11	0.52 (0.22–0.68)	0.44 ± 0.12	0.39 (0.32–0.70)	0.031 *
	Lymphocytes [10^3^/mm^3^]	2.35 ± 0.46	2.32 (1.50–3.14)	2.11 ± 0.44	1.97 (1.47–3.06)	0.110
Immunophenotype	T lymphocytes CD3+ [%]	72.67 ± 2.95	73.36 (66.16–79.79)	71.65 ± 3.17	71.98 (66.17–79.40)	0.156
	B lymphocytes CD19+ [%]	11.14 ± 2.24	11.00 (8.45–16.14)	11.19 ± 2.51	10.05 (8.21–16.30)	0.935
	NK cells CD3-CD16+CD56+ [%]	10.58 ± 2.28	10.22 (6.11–14.63)	13.34 ± 2.39	13.12 (7.05–17.24)	0.0001 *
	NKT-like cells CD3+CD16+CD56+ [%]	2.03 ± 1.44	1.44 (0.57–5.63)	3.61 ± 1.74	3.33 (0.79–6.55)	0.004 *
	T lymphocytes CD3+CD4+ [%]	40.25 ± 3.28	40.09 (34.53–47.08)	38.82 ± 2.36	38.18 (35.68–42.17)	0.173
	T lymphocytes CD3+CD8+ [%]	27.17 ± 3.44	26.59 (22.66–34.25)	31.24 ± 3.35	30.53 (22.28–36.44)	0.001 *
	T lymphocytes ratio CD3+CD4+/T CD3+CD8+	1.51 ± 0.26	1.49 (1.05–2.02)	1.26 ± 0.19	1.27 (1.02–1.85)	0.000 *

* Statistically significant results.

**Table 3:** Comparison of selected parameters of peripheral blood of GC patients with regard to grade.

Parameters		G1 (*n* = 10)		G2 (*n* = 13)		G3 (*n* = 17)		*p*-Value	*p*-Value		
		Mean ± SD	Median (Range)	Mean ± SD	Median (Range)	Mean ± SD	Median (Range)		G1 vs. G2	G1 vs. G3	G2 vs. G3
Morphology	White blood cells [10^3^/mm^3^]	6.75 ± 0.60	6.46 (6.06–7.70)	7.68 ± 0.88	7.48 (6.30–9.57)	8.57 ± 1.21	8.87 (5.99–10.20)	0.002 *	0.049 *	0.001 *	0.038 *
	Monocytes [10^3^/mm^3^]	0.43 ± 0.12	0.39 (0.33–0.70)	0.45 ± 0.11	0.42 (0.32–0.66)	0.53 ± 0.11	0.55 (0.22–0.68)	0.047 *	0.927	0.001 *	0.071
	Lymphocytes [10^3^/mm^3^]	2.13 ± 0.42	2.00 (1.47–3.05)	2.00 ± 0.45	1.89 (1.50–3.06)	2.50 ± 0.37	2.47 (1.84–3.124)	0.006 *	0.256	0.030 *	0.003 *
Immunophenotype	T lymphocytes CD3+ [%]	71.27 ± 2.38	72.15 (66.80–73.99)	72.37 ± 3.21	72.74 (66.17–79.40)	72.69 ± 3.23	73.36 (66.16–79.79)	0.354	0.483	0.127	0.681
	B lymphocytes CD19+ [%]	10.95 ± 2.83	9.76 (8.21–16.30)	10.71 ± 1.79	9.84 (8.45–14.27)	11.63 ± 2.35	11.18 (8.52–16.14)	0.549	0.692	0.386	0.408
	NK cells CD3-CD16+CD56+ [%]	14.41 ± 1.92	14.35 (11.75–17.24)	11.93 ± 1.88	12.27 (7.05–14.56)	10.07 ± 2.29	10.14 (6.11–14.63)	0.001 *	0.025 *	0.000 *	0.022 *
	NKT-like cells CD3+CD16+CD56+ [%]	4.41 ± 1.67	4.96 (2.06–6.55)	2.53 ± 1.03	2.63 (0.79–4.20)	1.82 ± 1.53	1.24 (0.57–5.63)	0.011 *	0.021 *	0.001 *	0.058
	T lymphocytes CD3+CD4+ [%]	37.43 ± 1.86	36.91 (35.68–42.10)	40.29 ± 2.70	40.59 (36.13–46.59)	40.44 ± 3.14	40.41 (34.53–47.08)	0.018 *	0.009 *	0.011 *	0.836
	T lymphocytes CD3+CD8+ [%]	32.03 ± 2.36	31.73 (28.58–34.90)	29.56 ± 3.58	30.46 (22.28–36.44)	26.56 ± 3.48	26.16 (22.66–34.25)	0.002 *	0.186	0.001 *	0.04 *
	T lymphocytes ratio CD3+CD4+/T CD3+CD8+	1.18 ± 0.13	1.14 (1.02–1.42)	1.38 ± 0.20	1.33 (1.13–1.85)	1.55 ± 0.27	1.55 (1.05–2.02)	0.001 *	0.01 *	0.001 *	0.05 *

* Statistically significant results.

**Table 4:** Comparison of selected peripheral blood parameters of patients with GC, taking into account the TNM stage.

Parameters		TNM 1 (*n* = 7)		TNM 2 (*n* = 12)		TNM 3 (*n* = 13)		TNM 4 (*n* = 8)		*p*-Value	*p*-Value					
		Mean ± SD	Median (Range)	Mean ± SD	Median (Range)	Mean ± SD	Median (Range)	Mean ± SD	Median (Range)		1 vs. 2	1 vs. 3	1 vs. 4	2 vs. 3	2 vs. 4	3 vs. 4
Morphology	White blood cells [10^3^/mm^3^]	6.89 ± 0.67	6.49 (6.06–7.70)	7.56 ± 1.22	7.44 (6.36–10.20)	7.73 ± 1.07	7.57 (5.99–9.57)	9.18 ± 0.52	9.15 (8.22–10.12)	0.004 *	0.299	0.210	0.001 *	0.728	0.001 *	0.003 *
	Monocytes [10^3^/mm^3^]	0.46 ± 0.13	0.39 (0.33–0.70)	0.45 ± 0.11	0.42 (0.33–0.68)	0.46 ± 0.12	0.49 (0.22–0.66)	0.58 ± 0.06	0.58 (0.46–0.67)	0.06	0.967	0.816	0.120	0.611	0.012 *	0.015 *
	Lymphocytes [10^3^/mm^3^]	2.14 ± 0.49	1.98 (1.47–3.05)	2.20 ± 0.41	2.06 (1.70–3.14)	2.08 ± 0.44	1.97 (1.50–3.06)	2.69 ± 0.25	2.69 (2.25–3.10)	0.02 *	0.650	0.816	0.028 *	0.437	0.015 *	0.002 *
Immunophenotype	T lymphocytes CD3+ [%]	71.11 ± 2.41	71.98 (66.80–73.99)	71.85 ± 2.72	72.65 (66.16–75.30)	72.54 ± 3.01	73.24 (66.17–79.40)	73.29 ± 3.76	74.42 (66.17–79.79)	0.399	0.430	0.241	0.151	0.728	0.520	0.238
	B lymphocytes CD19+ [%]	10.87 ± 2.81	9.46 (8.21–16.30)	11.08 ± 2.38	9.95 (8.24–15.07)	11.02 ± 1.53	11.00 (8.82–14.27)	11.75 ± 2.87	11.62 (8.52–16.14)	0.871	0.530	0.588	0.612	0.728	0.678	0.859
	NK cells CD3-CD16+CD56+ [%]	14.04 ± 2.10	13.79 (11.75–7.24)	12.21 ± 2.78	12.50 (6.11–16.15)	11.78 ± 2.07	12.06 (7.05–14.63)	9.04 ± 1.29	9.29 (6.72–11.06)	0.002 *	0.340	0.080	0.001 *	0.538	0.009 *	0.002 *
	NKT-like cells CD3+CD16+CD56+ [%]	3.91 ± 1.76	3.62 (2.06–6.55)	3.28 ± 1.83	2.97 (0.57–6.05)	2.53 ± 1.38	2.38 (0.78–5.63)	1.04 ± 0.30	0.96 (0.67–1.72)	0.003 *	0.530	0.096	0.001 *	0.376	0.009 *	0.004 *
	T lymphocytes CD3+CD4+ [%]	37.49 ± 2.19	36.13 (35.68–42.10)	40.39 ± 2.71	39.81 (36.72–46.59)	39.39 ± 2.37	40.59 (35.90–42.32)	40.80 ± 3.81	40.23 (34.53–47.08)	0.143	0.070	0.096	0.120	0.437	0.734	0.696
	T lymphocytes CD3+CD8+ [%]	31.61 ± 2.38	30.27 (28.58–34.90)	29.55 ± 3.78	30.15 (22.66–34.87)	29.39 ± 3.87	30.46 (22.28–36.44)	24.77 ± 1.67	24.10 (22.80–28.08)	0.007 *	0.299	0.311	0.001 *	0.810	0.015 *	0.005 *
	T lymphocytes ratio CD3+CD4+/T CD3+CD8+	1.20 ± 0.15	1.19 (1.02–1.42)	1.40 ± 0.27	1.31 (1.06–1.89)	1.37 ± 0.22	1.35 (1.05–1.856)	1.66 ± 0.23	1.61 (1.42–2.02)	0.005 *	0.119	0.114	0.001 *	0.936	0.031 *	0.007 *

* Statistically significant results.

**Table 5:** Analysis of the percentage of TLR-9 on selected subpopulations of T and B lymphocytes in GC patients in relation to healthy volunteers.

Parameters	GC Patients (*n* = 40)		HV Patients (*n* = 30)		*p*-Value
	Mean ± SD	Median (Range)	Mean ± SD	Median (Range)	
T lymphocytes CD4+TLR-9+ [%]	8.78 ± 4.92	7.78 (2.65–19.86)	1.04 ± 0.56	0.99 (0.19–2.28)	0.000 *
T lymphocytes CD8+TLR-9+ [%]	8.76 ± 4.20	7.12 (3.32–16.72)	1.30 ± 0.57	1.41 (0.15–2.31)	0.000 *
B lymphocytes CD19+TLR-9+ [%]	12.84 ± 7.20	9.47 (5.38–29.57)	1.80 ± 0.78	1.63 (0.16–3.68)	0.000 *

* Statistically significant results.

**Table 6:** Analysis of the percentage of TLR-9 on selected subpopulations of T and B lymphocytes in patients with GC, taking into account Lauren classification.

Parameters	Diffuse-Type GC Patients (*n* = 23)		Intestinal GC Patients (*n* = 17)		*p*-Value
	Mean ± SD	Median (Range)	Mean ± SD	Median (Range)	
T lymphocytes CD4+TLR-9+ [%]	9.82 ± 5.33	9.41 (2.65–19.86)	7.38 ± 3.90	7.03 (2.72–15.35)	0.191
T lymphocytes CD8+TLR-9+ [%]	9.06 ± 4.32	7.63 (3.32–16.69)	8.36 ± 3.99	6.91 (4.84–16.72)	0.704
B lymphocytes CD19+TLR-9+ [%]	14.17 ± 6.87	13.02 (5.38–27.52)	11.03 ± 7.26	7.46 (5.46–29.57)	0.054

**Table 7:** Analysis of the percentage of TLR-9 on selected subpopulations of T and B lymphocytes in GC patients by grade.

Parameters	G1 (*n* = 20)		G2 (*n* = 9)		G3 (*n* = 11)		*p*-Value	*p*-Value		
	Mean ± SD	Median (Range)	Mean ± SD	Median (Range)	Mean ± SD	Median (Range)		G1 vs. G2	G1 vs. G3	G2 vs. G3
T lymphocytes CD4+TLR-9+ [%]	5.80 ± 2.32	5.63 (2.72–9.35)	8.87 ± 4.85	10.08 (2.65–15.35)	10.47 ± 5.26	9.41 (2.80–19.86)	0.082	0.231	0.035 *	0.245
T lymphocytes CD8+TLR-9+ [%]	6.59 ± 1.43	6.89 (4.93–8.85)	10.95 ± 4.99	14.11 (4.17–16.72)	8.37 ± 3.83	7.03 (3.32–15.29)	0.149	0.101	0.443	0.133
B lymphocytes CD19+TLR-9+ [%]	7.58 ± 1.45	7.43 (5.52–9.59)	15.18 ± 8.25	15.01 (6.31–29.57)	14.14 ± 6.83	13.02 (5.38–26.91)	0.027 *	0.03 *	0.001 *	0.967

* Statistically significant results.

**Table 8:** Analysis of the percentage of TLR-9 on selected subpopulations of T and B lymphocytes in patients with GC, taking into account the TNM stage.

Parameters	TNM I (*n* = 7)		TNM II (*n* = 12)		TNM III (*n* = 13)		TNM IV (*n* = 8)		*p*-Value	*p*-Value					
	Mean ± SD	Median (Range)	Mean ± SD	Median (Range)	Mean ± SD	Median (Range)	Mean ± SD	Median (Range)		1 vs. 2	1 vs. 3	1 vs. 4	2 vs. 3	2 vs. 4	3 vs. 4
T lymphocytes CD4+TLR-9+ [%]	6.11 ± 1.86	6.02 (3.32–8.69)	5.13 ± 2.70	3.19 (2.65–9.41)	10.75 ± 4.32	12.62 (2.80–15.35)	13.42 ± 4.77	15.43 (4.48–19.86)	0.0009 *	0.299	0.045 *	0.013 *	0.002 *	0.0001 *	0.063
T lymphocytes CD8+TLR-9+ [%]	6.96 ± 1.41	6.91 (4.93–8.85)	5.21 ± 1.49	4.99 (3.32–8.92)	12.27 ± 4.43	14.60 (3.79–16.72)	9.98 ± 2.82	10.56 (6.23–14.35)	0.0004 *	0.055	0.029 *	0.09	0.000 *	0.000 *	0.104
B lymphocytes CD19+TLR-9+ [%]	8.15 ± 1.26	8.36 (6.15–9.59)	7.12 ± 1.25	7.24 (5.38–9.77)	18.89 ± 7.37	17.52 (6.77–29.57)	15.66 ± 5.63	15.14 (7.53–26.61)	0.0001 *	0.167	0.003 *	0.003 *	0.000 *	0.000 *	0.268

* Statistically significant results.

**Table 9:** Analysis of selected parameters of GC patients, taking into account their division into GC EBV+ and GC EBV−.

Parametrs	GC EBV+		GC EBV−		*p*-Value
	Mean ± SD	Median (Range)	Mean ± SD	Median (Range)	
Age	61.32 ± 10.34	63.00 (40.00–77.00)	63.95 ± 10.52	64.00 (41.00–81.00)	0.485
Sex	Female: 36.84% Male: 63.16%		Female: 42.86% Male: 57.14%		NA
Lauren classification	Diffuse type: 78.95% Intestinal type: 21.05%		Diffuse type: 38.09% Intestinal type: 61.91%		NA
Grade	G1: 0.00% G2: 36.84% G3:63.16%		G1: 47.62% G2: 28.57% G3:23.81%		NA
TNM stage	TNM I: 0.00% TNM II: 0.00% TNM III: 57.90% TNM IV: 42.10%		TNM I: 33.33% TNM II: 57.14% TNM III: 9.53% TNM IV: 0.00%		NA
EBV copy number	570.74 ± 503.12	451.78 (79.35–1927.36)	NA		NA
White blood cells [10^3^/mm^3^]	8.45 ± 1.05	8.78 (6.30–10.12)	7.26 ± 1.09	7.39 (5.99–10.20)	0.0002 *
Monocytes [10^3^/mm^3^]	0.52 ± 0.11	0.55 (0.22–0.67)	0.44 ± 0.11	0.40 (0.32–0.70)	0.029 *
Lymphocytes [10^3^/mm^3^]	2.37 ± 0.46	2.43 (1.50–3.10)	2.13 ± 0.44	1.98 (1.47–3.14)	0.100
T lymphocytes CD3+ [%]	72.82 ± 3.51	73.48 (66.17–79.79)	71.70 ± 2.54	72.39 (66.16–75.30)	0.258
B lymphocytes CD19+ [%]	11.25 ± 2.26	11.03 (8.52–16.14)	11.07 ± 2.44	10.05 (8.21–16.30)	0.668
NK cells CD3-CD16+CD56+ [%]	10.42 ± 2.13	10.16 (6.72–14.63)	12.96 ± 2.59	12.78 (6.11–17.24)	0.001 *
NKT-like cells CD3+CD16+CD56+ [%]	1.78 ± 1.24	1.24 (0.67–5.63)	3.53 ± 1.74	3.33 (0.57–6.55)	0.001 *
T lymphocytes CD3+CD4+ [%]	39.77 ± 3.19	40.05 (34.53–47.08)	39.53 (2.873)	39.08 (35.68–46.59)	0.872
T lymphocytes CD3+CD8+ [%]	27.24 ± 3.92	26.40 (22.28–36.44)	30.40 ± 3.33	30.27 (22.66–34.90)	0.009 *
T lymphocytes ratio CD3+CD4+/T CD3+CD8+	1.49 ± 0.27	1.46 (1.05–2.02)	1.33 ± 0.24	1.29 (1.02–1.89)	0.003 *
T lymphocytes CD4+TLR-9+ [%]	12.64 ± 4.01	14.13 (4.48–19.86)	5.29 ± 2.43	4.22 (2.65–9.41)	0.000 *
T lymphocytes CD8+TLR-9+ [%]	12.12 ± 3.55	12.58 (6.23–16.72)	5.73 ± 1.67	5.09 (3.32–8.92)	0.000 *
B lymphocytes CD19+TLR-9+ [%]	18.77 ± 6.36	17.49 (7.53–29.57)	7.47 ± 1.29	7.39 (5.38–9.77)	0.000 *
TLR-9 serum concentration [ng/mL]	23.13 ± 4.44	23.38 (15.51–31.70)	9.44 ± 3.17	9.47 (4.99–17.30)	0.000 *

* Statistically significant results.

**Table 10:** Selected parameters of GC patients with regard to survival status.

Parametrs	Dead GC Patients		Alive GC Patients		*p*-Value
	Mean ± SD	Median (Range)	Mean ± SD	Median (Range)	
Age	61.96 ± 10.20	63.00 (40.00–77.00)	62.23 ± 11.00	67.00 (41.00–81.00)	0.487
Sex	Female: 33.33% Male: 66.67%		Female: 53.85% Male: 46.15%		NA
Lauren classification	Diffuse type: 91.31% Intestinal type: 8.69%		Diffuse type: 35.29% Intestinal type: 64.71%		NA
Grade	G1: 0.00% G2: 40.74% G3: 59.26%		G1: 76.92% G2: 15.38% G3: 7.70%		NA
TNM stage	TNM I: 0.00% TNM II: 25.92% TNM III: 48.15% TNM IV: 25.93%		TNM I: 53.85% TNM II: 38.45% TNM III: 0.00% TNM IV: 0.00%		NA
EBV copy number	570.74 ± 503.12	451.78 (79.35–1927.86)	NA		NA
White blood cells [10^3^/mm^3^]	8.30 ± 1.13	8.39 (5.99–10.20)	6.83 ± 0.69	6.47 (6.06–8.33)	0.000 *
Monocytes [10^3^/mm^3^]	0.49 ± 0.12	0.52 (0.22–0.68)	0.45 ± 0.11	0.40 (0.33–0.70)	0.206
Lymphocytes [10^3^/mm^3^]	2.29 ± 0.50	2.29 (1.50–3.14)	2.15 ± 0.38	2.02 (1.47–3.05)	0.493
T lymphocytes CD3+ [%]	72.27 ± 3.32	72.90 (66.16–79.79)	71.95 ± 2.51	72.39 (66.80–75.30)	0.753
B lymphocytes CD19+ [%]	11.27 ± 2.13	11.03 (8.52–16.14)	10.93 ± 2.76	9.84 (8.21–16.30)	0.407
NK cells CD3-CD16+CD56+ [%]	10.69 ± 2.30	10.90 (6.11–14.63)	13.98 ± 2.03	13.89 (10.36–17.24)	0.000 *
NKT-like cells CD3+CD16+CD56+ [%]	1.90 ± 1.25	1.44 (0.57–5.63)	4.23 ± 1.67	4.71 (1.53–6.55)	0.000 *
T lymphocytes CD3+CD4+ [%]	40.15 ± 2.84	40.41 (34.53–47.08)	38.58 ± 3.08	37.93 (35.68–46.59)	0.056
T lymphocytes CD3+CD8+ [%]	27.50 ± 3.77	27.05 (22.28–36.44)	31.81 ± 2.44	32.89 (27.46–34.90)	0.001 *
T lymphocytes ratio CD3+CD4+/T CD3+CD8+	1.49 ± 0.26	1.43 (1.05–2.02)	1.23 ± 0.18	1.19 (1.02–1.70)	0.001 *
T lymphocytes CD4+TLR-9+ [%]	10.41 ± 5.03	11.47 (2.65–19.86)	5.41 ± 2.29	5.23 (2.72–9.35)	0.005 *
T lymphocytes CD8+TLR-9+ [%]	9.98 ± 4.58	10.02 (3.32–16.72)	6.44 ± 1.64	6.86 (4.05–8.92)	0.005 *
B lymphocytes CD19+TLR-9+ [%]	15.29 ± 7.57	15.01 (5.38–29.57)	7.73 ± 1.40	7.46 (5.52–9.77)	0.003 *
TLR-9 serum concentration [ng/mL]	19.42 ± 6.94	19.87 (5.74–31.70)	8.73 ± 3.42	7.65 (4.99–17.30)	0.000 *

* Statistically significant results.

## Data Availability

All necessary information regarding this publication is available upon written request from the first author of this publication.

## Supplementary Materials

The following supporting information can be downloaded at: https://www.mdpi.com/article/10.3390/cancers15205104/s1. Figure S1: Analysis of the percentage of TLR-9 on selected subpopulations of T (A, B) and B (C) lymphocytes in GC patients according to Lauren classification. Patients with diffuse type were marked in purple color and intestinal type in blue. Figure S2: Analysis of the percentage of TLR-9 on selected subpopulations of T cells (A, B) and B (C) in GC patients by grade. For easier interpretation of the results, individual grades are marked with colors. Statistically significant results are marked *. Figure S3: Analysis of the percentage of TLR-9 on selected subpopulations of T (A, B) and B (C) lymphocytes in GC patients by TNM stage. For easier interpretation of the results, individual grades are marked with colors. Statistically significant results are marked *. Table S1: Details of TLR-9 serum concentration [ng/mL]. Table S2: Spearman’s *rank* correlation analysis in Alive GC patients. Table S3: Sperman’s rank correlation analysis in Dead GC patients. Table S4: Sperman’s rank correlation analysis in GC Diffuse type patients. Table S5: Sperman’s rank correlation analysis in patients with GC Intestinal type. Table S6: Sperman’s rank correlation analysis in GC-G1 patients. Table S7: Sperman’s rank correlation analysis in GC-G2 patients. Table S8: Sperman’s rank correlation analysis in GC-G3 patients. Table S9: Sperman’s rank correlation analysis in GC-TNM I patients. Table S10: Sperman’s rank correlation analysis in GC-TNM II patients. Table S11: Sperman’s rank correlation analysis in GC-TNM III patients. Table S12: Sperman’s rank correlation analysis in GC-TNM IV patients.
