# Supplementary material for: How Toll-like Receptor 9 Plays a Key Role in the Development of Gastric Cancer and Is Linked to Epstein–Barr Virus Infection
Source: Cancers (Basel). 2023 Oct 23;15(20):5104. doi: 10.3390/cancers15205104 (PMC10605881; doi:10.3390/cancers15205104)
Supplement: Supplementary file 1 [file cancers-15-05104-s001.zip › cancers-2622388-supplementary.pdf]

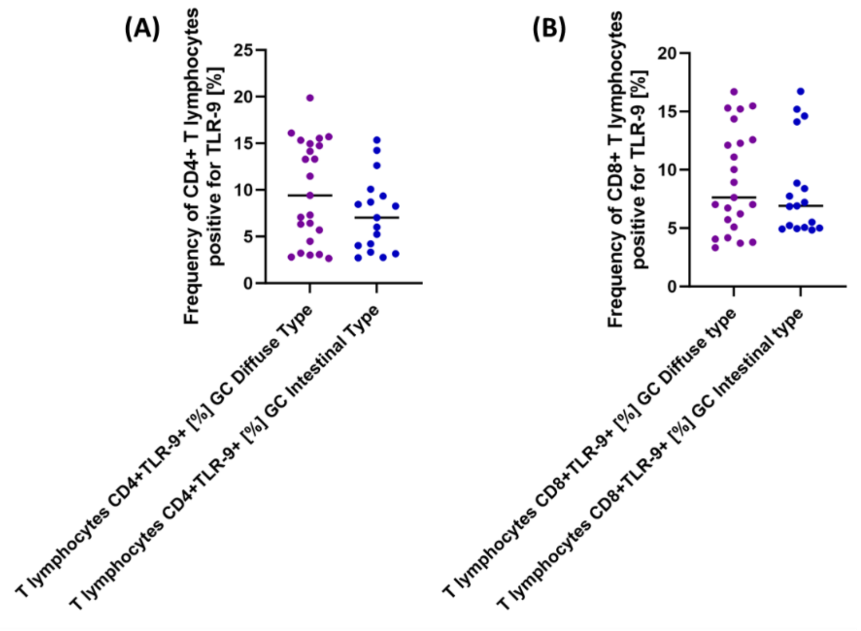

Supplementary Materials Figure 1. Analysis of the percentage of TLR-9 on selected subpopulations of T (A, B) and B (C) lymphocytes in GC patients according to Lauren classification. Patients with diffuse type were marked in purple color and intestinal type in blue.

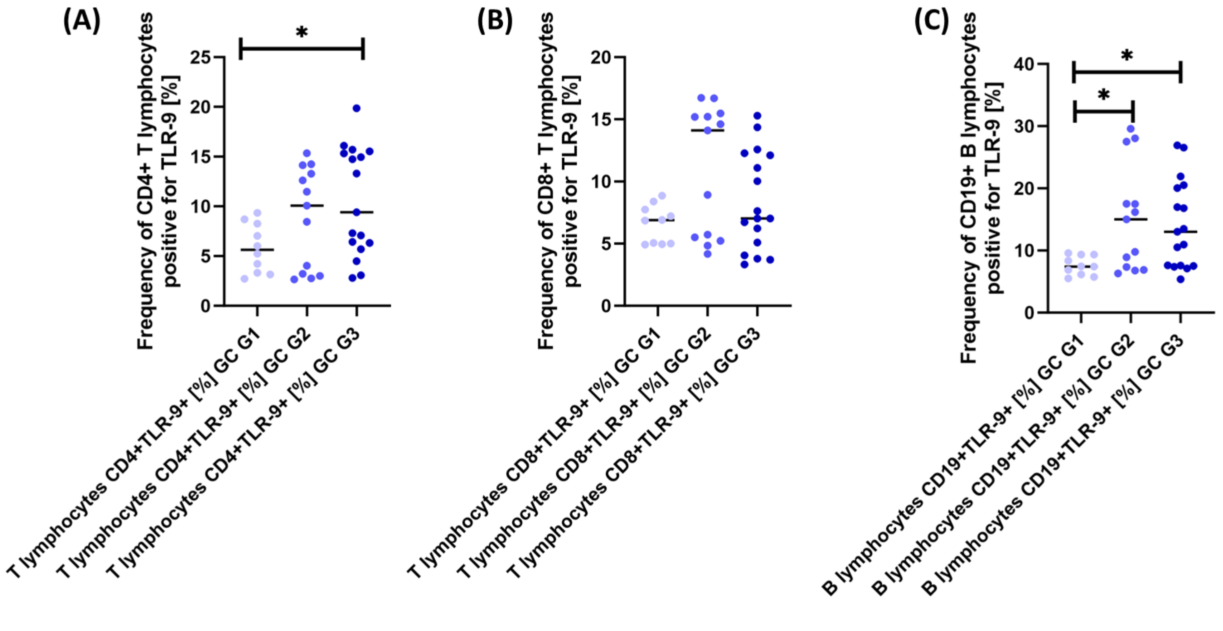

Supplementary Materials Figure 2. Analysis of the percentage of TLR-9 on selected subpopulations of T cells (A, B) and B (C) in GC patients by grade. For easier interpretation of the results, individual grades are marked with colors. Statistically significant results are marked *.

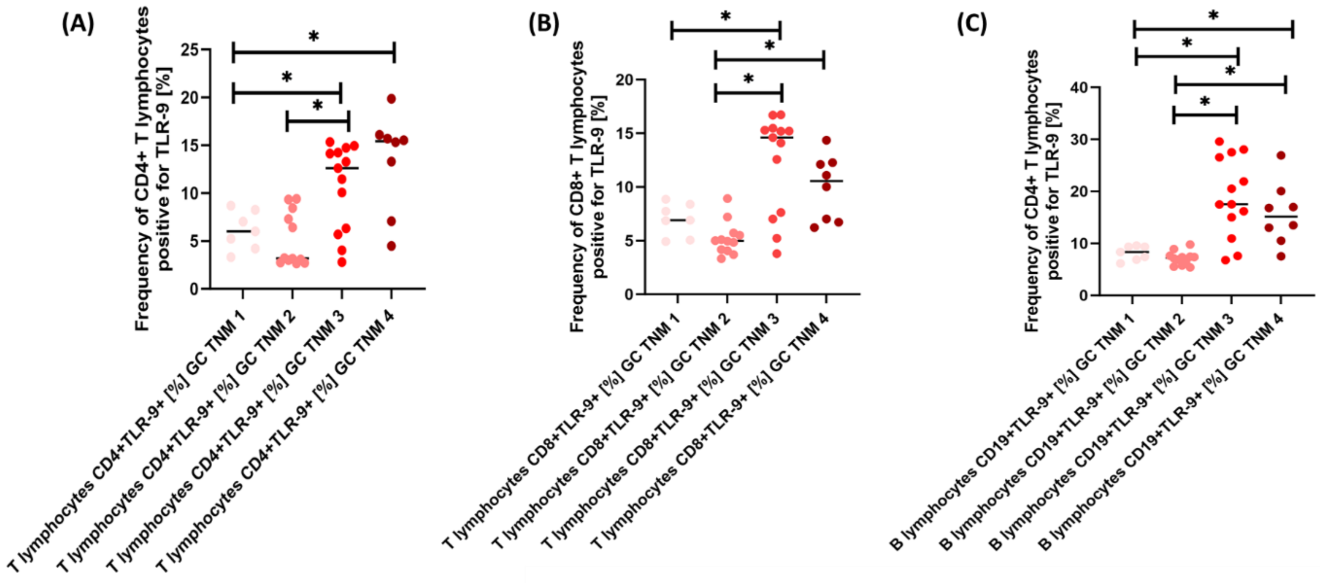

Supplementary Materials Figure 3. Analysis of the percentage of TLR-9 on selected subpopulations of T (A, B) and B (C) lymphocytes in GC patients by TNM stage. For easier interpretation of the results, individual grades are marked with colors. Statistically significant results are marked *.

Supplementary Materials Table S1: Details of TLR-9 serum concentration [ng/mL].

**Parameters**			**TLR-9 serum concentration [ng/mL]**	
**Total**	GC patients	Mean±SD	15.95± 7.83	0.000*
		Median (range)	14.09 (4.99-31.70)	
	HV	Mean±SD	3.05± 0.57	
		Median (range)	3.16 (2.03-3.92)	
**Lauren classification**	Diffuse type	Mean±SD	18.10± 7.44	0.036*
		Median (range)	18.09 (5.74-31.70)	
	Intestinal type	Mean±SD	13.04± 7.26	
		Median (range)	11.46 (4.99-28.83)	
**Grade**	G1	Mean±SD	8.70± 3.76	p- value: 0,0001*<br>G1 vs. G2 0.001*<br>G1 vs. G3 0.001*<br>G2 vs .G3 0.869
		Median (range)	8.06 (4.99-17.30)	
	G2	Mean±SD	18.45± 6.98	
		Median (range)	19.87 (7.65-28.83)	
	G3	Mean±SD	18.30± 7.59	
		Median (range)	18.09 (5.74-31.70)	
**TNM stage**	TNM I	Mean±SD	7.78± 2.73	p- value: 0,000*<br>I vs II: 0.142<br>I vs. III: 0.000*<br>I vs. IV: 0.003*<br>II vs. III: 0.000*<br>II vs. IV: 0.000*<br>III vs. IV: 0.645
		Median (range)	6.65 (4.99-11.80)	
	TNM II	Mean±SD	10.02± 3.22	
		Median (range)	9.45 (5.71-17.30)	
	TNM III	Mean±SD	22.47± 6.30	
		Median (range)	22.97 (11.72-31.70)	
	TNM IV	Mean±SD	21.38± 3.25	
		Median (range)	23.22 (15.51-24.94)	

Supplementary Materials Table S2: Spearman's *rank* correlation analysis in Alive GC patients.

**Parameters**	**R**	**t(N-2)**	**p**
**T lymphocytes CD3+CD8+ [%] & T lymphocytes ratio CD3+CD4+/T CD3+CD8+**	-0.929	-8.298	0.000*
**NKT-like cells CD3+CD16+CD56+ [%] & T lymphocytes ratio CD3+CD4+/T CD3+CD8+**	-0.896	-6.677	0.000*
**White blood cells [10^3/mm^3] & NKT-like cells CD3+CD16+CD56+ [%]**	-0.786	-4.213	0.001*

NK cells CD3-CD16+CD56+ [%] & T lymphocytes ratio CD3+CD4+/T CD3+CD8+	-0.769	-3.993	0.002*
T lymphocytes CD3+CD4+ [%] & T lymphocytes CD3+CD8+ [%]	-0.764	-3.924	0.002*
NK cells CD3-CD16+CD56+ [%] & T lymphocytes CD3+CD4+ [%]	-0.692	-3.182	0.009*
White blood cells [10^3/mm^3] & NK cells CD3-CD16+CD56+ [%]	-0.659	-2.909	0.014**
NKT-like cells CD3+CD16+CD56+ [%] & T lymphocytes CD3+CD4+ [%]	-0.659	-2.909	0.014*
NK cells CD3-CD16+CD56+ [%] & Survival time from diagnosis [months]	-0.566	-2.277	0.044*
White blood cells [10^3/mm^3] & T lymphocytes ratio CD3+CD4+/T CD3+CD8+	0.604	2.516	0.029*
White blood cells [10^3/mm^3] & Survival time from diagnosis [months]	0.632	2.704	0.021*
NK cells CD3-CD16+CD56+ [%] & T lymphocytes CD3+CD8+ [%]	0.786	4.213	0.001*
NK cells CD3-CD16+CD56+ [%] & NKT-like cells CD3+CD16+CD56+ [%]	0.846	5.266	0.000*
T lymphocytes CD3+CD4+ [%] & T lymphocytes ratio CD3+CD4+/T CD3+CD8+	0.896	6.677	0.000*
NKT-like cells CD3+CD16+CD56+ [%] & T lymphocytes CD3+CD8+ [%]	0.901	6.892	0.000*

Supplementary Materials Table S3: Sperman's rank correlation analysis in Dead GC patients.

Parameters	R	t(N-2)	p
White blood cells [10^3/mm^3] & NK cells CD3-CD16+CD56+ [%]	-0.996	-58.256	0.000*
White blood cells [10^3/mm^3] & NKT-like cells CD3+CD16+CD56+ [%]	-0.994	-46.845	0.000*
Lymphocytes [10^3/mm^3] & NK cells CD3-CD16+CD56+ [%]	-0.946	-14.608	0.000*
T lymphocytes CD3+CD8+ [%] & T lymphocytes ratio CD3+CD4+/T CD3+CD8+	-0.937	-13.425	0.000*
Lymphocytes [10^3/mm^3] & NKT-like cells CD3+CD16+CD56+ [%]	-0.931	-12.705	0.000*
White blood cells [10^3/mm^3] & T lymphocytes CD3+CD8+ [%]	-0.896	-10.066	0.000*
NK cells CD3-CD16+CD56+ [%] & T lymphocytes ratio CD3+CD4+/T CD3+CD8+	-0.869	-8.796	0.000*
Lymphocytes [10^3/mm^3] & T lymphocytes CD3+CD8+ [%]	-0.866	-8.664	0.000*
Survival time from diagnosis [months] & DNA EBV copy number [copies/1 ug DNA]	-0.861	-8.451	0.000*
NKT-like cells CD3+CD16+CD56+ [%] & T lymphocytes ratio CD3+CD4+/T CD3+CD8+	-0.835	-7.596	0.000*
Monocytes [10^3/mm^3] & NKT-like cells CD3+CD16+CD56+ [%]	-0.828	-7.396	0.000*
Monocytes [10^3/mm^3] & NK cells CD3-CD16+CD56+ [%]	-0.823	-7.252	0.000*
Survival time from diagnosis [months] & TLR-9 serum concentration [ng/mL]	-0.791	-6.459	0.000*
Survival time from diagnosis [months] & T lymphocytes CD4+TLR-9+ [%]	-0.764	-5.929	0.000*
Survival time from diagnosis [months] & T lymphocytes CD8+TLR-9+ [%]	-0.764	-5.923	0.000*
Survival time from diagnosis [months] & B lymphocytes CD19+TLR-9+ [%]	-0.733	-5.391	0.000*
Monocytes [10^3/mm^3] & T lymphocytes CD3+CD8+ [%]	-0.708	-5.010	0.000*
Stage (TNM) [I-IV] & Survival time from diagnosis [months]	-0.484	-2.767	0.010*

**NKT-like cells CD3+CD16+CD56+ [%] & T lymphocytes CD3+CD4+ [%]**	-0.447	-2.498	0.019*
**Survival time from diagnosis [months] & T lymphocytes CD3+ [%]**	-0.433	-2.405	0.024*
**NK cells CD3-CD16+CD56+ [%] & T lymphocytes CD3+CD4+ [%]**	-0.420	-2.314	0.029*
**Monocytes [10^3/mm^3] & T lymphocytes CD3+ [%]**	0.392	2.129	0.043*
**T lymphocytes CD4+TLR-9+ [%] & T lymphocytes CD3+ [%]**	0.402	2.194	0.038*
**B lymphocytes CD19+TLR-9+ [%] & Stage (TNM) [I-IV]**	0.407	2.225	0.035*
**T lymphocytes CD3+CD4+ [%] & White blood cells [10^3/mm^3]**	0.424	2.343	0.027*
**DNA EBV copy number [copies/1 ug DNA] & Stage (TNM) [I-IV]**	0.454	2.549	0.017*
**TLR-9 serum concentration [ng/mL] & Stage (TNM) [I-IV]**	0.476	2.707	0.012*
**T lymphocytes CD4+TLR-9+ [%] & Stage (TNM) [I-IV]**	0.576	3.519	0.002*
**B lymphocytes CD19+TLR-9+ [%] & T lymphocytes CD4+TLR-9+ [%]**	0.602	3.769	0.001*
**T lymphocytes ratio CD3+CD4+/T CD3+CD8+ & T lymphocytes CD3+CD4+ [%]**	0.603	3.775	0.001*
**T lymphocytes CD4+TLR-9+ [%] & T lymphocytes CD8+TLR-9+ [%]**	0.667	4.472	0.000*
**T lymphocytes CD4+TLR-9+ [%] & TLR-9 serum concentration [ng/mL]**	0.712	5.068	0.000*
**DNA EBV copy number [copies/1 ug DNA] & T lymphocytes CD4+TLR-9+ [%]**	0.762	5.883	0.000*
**Monocytes [10^3/mm^3] & Lymphocytes [10^3/mm^3]**	0.779	6.208	0.000*
**White blood cells [10^3/mm^3] & Monocytes [10^3/mm^3]**	0.809	6.872	0.000*
**Lymphocytes [10^3/mm^3] & T lymphocytes ratio CD3+CD4+/T CD3+CD8+**	0.813	6.974	0.000*
**T lymphocytes CD8+TLR-9+ [%] & B lymphocytes CD19+TLR-9+ [%]**	0.825	7.310	0.000*
**B lymphocytes CD19+TLR-9+ [%] & TLR-9 serum concentration [ng/mL]**	0.827	7.344	0.000*
**White blood cells [10^3/mm^3] & T lymphocytes ratio CD3+CD4+/T CD3+CD8+**	0.856	8.276	0.000*
**NKT-like cells CD3+CD16+CD56+ [%] & T lymphocytes CD3+CD8+ [%]**	0.870	8.814	0.000*
**T lymphocytes CD8+TLR-9+ [%] & TLR-9 serum concentration [ng/mL]**	0.880	9.280	0.000*
**DNA EBV copy number [copies/1 ug DNA] & B lymphocytes CD19+TLR-9+ [%]**	0.890	9.759	0.000*
**DNA EBV copy number [copies/1 ug DNA] & T lymphocytes CD8+TLR-9+ [%]**	0.899	10.243	0.000*
**NK cells CD3-CD16+CD56+ [%] & T lymphocytes CD3+CD8+ [%]**	0.911	11.035	0.000*
**DNA EBV copy number [copies/1 ug DNA] & TLR-9 serum concentration [ng/mL]**	0.941	13.866	0.000*
**White blood cells [10^3/mm^3] & Lymphocytes [10^3/mm^3]**	0.952	15.483	0.000*
**NK cells CD3-CD16+CD56+ [%] & NKT-like cells CD3+CD16+CD56+ [%]**	0.991	36.383	0.000*

Supplementary Materials Table S4: Sperman's rank correlation analysis in GC Diffuse type patients.

**Parameters**	**R**	**t(N-2)**	**p**
**White blood cells [10^3/mm^3] & NK cells CD3-CD16+CD56+ [%]**	-0.996	-51.388	0.000*
**T lymphocytes CD3+CD8+ [%] & White blood cells [10^3/mm^3]**	-0.960	-15.812	0.000*

**Lymphocytes [10^3/mm^3] & NK cells CD3-CD16+CD56+ [%]**	-0.957	-15.165	0.000*
**NK cells CD3-CD16+CD56+ [%] & Lymphocytes [10^3/mm^3]**	-0.957	-15.165	0.000*
**NKT-like cells CD3+CD16+CD56+ [%] & Lymphocytes [10^3/mm^3]**	-0.947	-13.490	0.000*
**NKT-like cells CD3+CD16+CD56+ [%] & T lymphocytes ratio CD3+CD4+/T CD3+CD8+**	-0.870	-8.078	0.000*
**Survival time from diagnosis [months] & DNA EBV copy number [copies/1 ug DNA]**	-0.869	-8.045	0.000*
**DNA EBV copy number [copies/1 ug DNA] & Survival time from diagnosis [months]**	-0.869	-8.045	0.000*
**NK cells CD3-CD16+CD56+ [%] & T lymphocytes ratio CD3+CD4+/T CD3+CD8+**	-0.869	-8.032	0.000*
**T lymphocytes ratio CD3+CD4+/T CD3+CD8+ & NK cells CD3-CD16+CD56+ [%]**	-0.869	-8.032	0.000*
**Monocytes [10^3/mm^3] & NKT-like cells CD3+CD16+CD56+ [%]**	-0.827	-6.750	0.000*
**Survival time from diagnosis [months] & TLR-9 serum concentration [ng/mL]**	-0.800	-6.114	0.000*
**T lymphocytes CD3+CD8+ [%] & Monocytes [10^3/mm^3]**	-0.774	-5.601	0.000*
**Survival time from diagnosis [months] & T lymphocytes CD8+TLR-9+ [%]**	-0.724	-4.809	0.000*
**T lymphocytes CD8+TLR-9+ [%] & Survival time from diagnosis [months]**	-0.724	-4.809	0.000*
**Stage (TNM) [I-IV] & Survival time from diagnosis [months]**	-0.645	-3.871	0.001*
**T lymphocytes CD3+CD4+ [%] & T lymphocytes CD3+CD8+ [%]**	-0.502	-2.660	0.015*
**T lymphocytes CD3+CD8+ [%] & T lymphocytes CD3+CD4+ [%]**	-0.502	-2.660	0.015*
**NKT-like cells CD3+CD16+CD56+ [%] & T lymphocytes CD3+CD4+ [%]**	-0.479	-2.500	0.021*
**NK cells CD3-CD16+CD56+ [%] & T lymphocytes CD3+CD4+ [%]**	-0.477	-2.489	0.021*
**T lymphocytes CD3+CD4+ [%] & NK cells CD3-CD16+CD56+ [%]**	-0.477	-2.489	0.021*
**Stage (TNM) [I-IV] & Lymphocytes [10^3/mm^3]**	0.414	2.084	0.050*
**Lymphocytes [10^3/mm^3] & Stage (TNM) [I-IV]**	0.414	2.084	0.050*
**T lymphocytes CD3+ [%] & T lymphocytes CD8+TLR-9+ [%]**	0.420	2.121	0.046*
**T lymphocytes CD8+TLR-9+ [%] & T lymphocytes CD3+ [%]**	0.420	2.121	0.046*
**Lymphocytes [10^3/mm^3] & T lymphocytes CD3+CD4+ [%]**	0.424	2.146	0.044*
**White blood cells [10^3/mm^3] & T lymphocytes CD3+CD4+ [%]**	0.458	2.364	0.028*
**T lymphocytes CD4+TLR-9+ [%] & Lymphocytes [10^3/mm^3]**	0.466	2.410	0.025*
**Stage (TNM) [I-IV] & T lymphocytes CD8+TLR-9+ [%]**	0.470	2.443	0.023*
**Monocytes [10^3/mm^3] & T lymphocytes CD3+ [%]**	0.473	2.459	0.023*
**T lymphocytes CD3+ [%] & Monocytes [10^3/mm^3]**	0.473	2.459	0.023*
**B lymphocytes CD19+TLR-9+ [%] & Stage (TNM) [I-IV]**	0.519	2.780	0.011*

**Stage (TNM) [I-IV] & TLR-9 serum concentration [ng/mL]**	0.617	3.594	0.002*
**TLR-9 serum concentration [ng/mL] & Stage (TNM) [I-IV]**	0.617	3.594	0.002*
**T lymphocytes CD4+TLR-9+ [%] & Stage (TNM) [I-IV]**	0.642	3.835	0.001*
**Monocytes [10^3/mm^3] & T lymphocytes ratio CD3+CD4+/T CD3+CD8+**	0.648	3.903	0.001*
**T lymphocytes ratio CD3+CD4+/T CD3+CD8+ & Monocytes [10^3/mm^3]**	0.648	3.903	0.001*
**T lymphocytes CD4+TLR-9+ [%] & TLR-9 serum concentration [ng/mL]**	0.693	4.401	0.000*
**T lymphocytes CD4+TLR-9+ [%] & T lymphocytes CD8+TLR-9+ [%]**	0.694	4.413	0.000*
**White blood cells [10^3/mm^3] & Monocytes [10^3/mm^3]**	0.792	5.951	0.000*
**T lymphocytes ratio CD3+CD4+/T CD3+CD8+ & T lymphocytes CD3+CD4+ [%]**	0.796	6.036	0.000*
**Lymphocytes [10^3/mm^3] & T lymphocytes ratio CD3+CD4+/T CD3+CD8+**	0.817	6.502	0.000*
**T lymphocytes CD4+TLR-9+ [%] & DNA EBV copy number [copies/1 ug DNA]**	0.824	6.667	0.000*
**T lymphocytes CD8+TLR-9+ [%] & B lymphocytes CD19+TLR-9+ [%]**	0.845	7.237	0.000*
**B lymphocytes CD19+TLR-9+ [%] & T lymphocytes CD8+TLR-9+ [%]**	0.845	7.237	0.000*
**TLR-9 serum concentration [ng/mL] & T lymphocytes CD8+TLR-9+ [%]**	0.845	7.237	0.000*
**White blood cells [10^3/mm^3] & T lymphocytes ratio CD3+CD4+/T CD3+CD8+**	0.863	7.816	0.000*
**T lymphocytes ratio CD3+CD4+/T CD3+CD8+ & White blood cells [10^3/mm^3]**	0.863	7.816	0.000*
**B lymphocytes CD19+TLR-9+ [%] & DNA EBV copy number [copies/1 ug DNA]**	0.879	8.452	0.000*
**DNA EBV copy number [copies/1 ug DNA] & T lymphocytes CD8+TLR-9+ [%]**	0.886	8.741	0.000*
**DNA EBV copy number [copies/1 ug DNA] & TLR-9 serum concentration [ng/mL]**	0.958	15.277	0.000*
**TLR-9 serum concentration [ng/mL] & DNA EBV copy number [copies/1 ug DNA]**	0.958	15.277	0.000*
**T lymphocytes CD3+CD8+ [%] & NK cells CD3-CD16+CD56+ [%]**	0.962	16.248	0.000*

Supplementary Materials Table S5: Sperman's rank correlation analysis in patients with GC Intestinal type.

**Parameters**	**R**	**t(N-2)**	**p**
**White blood cells [10^3/mm^3] & NKT-like cells CD3+CD16+CD56+ [%]**	-0.929	-9.716	0.000*
**White blood cells [10^3/mm^3] & NK cells CD3-CD16+CD56+ [%]**	-0.892	-7.649	0.000*
**Survival time from diagnosis [months] & TLR-9 serum concentration [ng/mL]**	-0.846	-6.135	0.000*
**T lymphocytes CD3+CD8+ [%] & T lymphocytes ratio CD3+CD4+/T CD3+CD8+**	-0.824	-5.623	0.000*

Survival time from diagnosis [months] & DNA EBV copy number [copies/1 ug DNA]	-0.741	-4.273	0.001*
Stage (TNM) [I-IV] & Survival time from diagnosis [months]	-0.723	-4.050	0.001*
NK cells CD3-CD16+CD56+ [%] & T lymphocytes CD8+TLR-9+ [%]	-0.716	-3.969	0.001*
NKT-like cells CD3+CD16+CD56+ [%] & T lymphocytes ratio CD3+CD4+/T CD3+CD8+	-0.679	-3.581	0.003*
Survival time from diagnosis [months] & T lymphocytes CD4+TLR-9+ [%]	-0.647	-3.287	0.005*
NK cells CD3-CD16+CD56+ [%] & T lymphocytes ratio CD3+CD4+/T CD3+CD8+	-0.645	-3.266	0.005*
NKT-like cells CD3+CD16+CD56+ [%] & T lymphocytes CD8+TLR-9+ [%]	-0.640	-3.223	0.006*
NKT-like cells CD3+CD16+CD56+ [%] & T lymphocytes CD3+CD4+ [%]	-0.623	-3.081	0.008*
NK cells CD3-CD16+CD56+ [%] & T lymphocytes CD3+CD4+ [%]	-0.618	-3.042	0.008*
DNA EBV copy number [copies/1 ug DNA] & NK cells CD3-CD16+CD56+ [%]	-0.553	-2.572	0.021*
NK cells CD3-CD16+CD56+ [%] & DNA EBV copy number [copies/1 ug DNA]	-0.553	-2.572	0.021*
Survival time from diagnosis [months] & B lymphocytes CD19+TLR-9+ [%]	-0.551	-2.560	0.022*
NK cells CD3-CD16+CD56+ [%] & T lymphocytes CD4+TLR-9+ [%]	-0.527	-2.401	0.030*
NK cells CD3-CD16+CD56+ [%] & B lymphocytes CD19+TLR-9+ [%]	-0.525	-2.386	0.031*
NK cells CD3-CD16+CD56+ [%] & T lymphocytes CD3+CD8+ [%]	0.490	2.178	0.046*
White blood cells [10^3/mm^3] & T lymphocytes CD8+TLR-9+ [%]	0.515	2.325	0.035*
Monocytes [10^3/mm^3] & Lymphocytes [10^3/mm^3]	0.528	2.410	0.029*
White blood cells [10^3/mm^3] & T lymphocytes ratio CD3+CD4+/T CD3+CD8+	0.529	2.417	0.029*
B lymphocytes CD19+ [%] & T lymphocytes ratio CD3+CD4+/T CD3+CD8+	0.537	2.464	0.026*
T lymphocytes CD3+CD8+ [%] & NKT-like cells CD3+CD16+CD56+ [%]	0.537	2.464	0.026*
White blood cells [10^3/mm^3] & T lymphocytes CD3+CD4+ [%]	0.539	2.480	0.026*
DNA EBV copy number [copies/1 ug DNA] & White blood cells [10^3/mm^3]	0.553	2.572	0.021*
Stage (TNM) [I-IV] & T lymphocytes CD3+CD4+ [%]	0.587	2.809	0.013*
T lymphocytes CD3+ [%] & T lymphocytes CD3+CD8+ [%]	0.630	3.141	0.007*
Stage (TNM) [I-IV] & DNA EBV copy number [copies/1 ug DNA]	0.715	3.963	0.001*
DNA EBV copy number [copies/1 ug DNA] & T lymphocytes CD4+TLR-9+ [%]	0.721	4.032	0.001*
T lymphocytes CD4+TLR-9+ [%] & B lymphocytes CD19+TLR-9+ [%]	0.730	4.142	0.001*
T lymphocytes CD8+TLR-9+ [%] & B lymphocytes CD19+TLR-9+ [%]	0.735	4.202	0.001*

DNA EBV copy number [copies/1 ug DNA] & T lymphocytes CD8+TLR-9+ [%]	0.741	4.273	0.001*
DNA EBV copy number [copies/1 ug DNA] & TLR-9 serum concentration [ng/mL]	0.741	4.273	0.001*
B lymphocytes CD19+TLR-9+ [%] & DNA EBV copy number [copies/1 ug DNA]	0.741	4.273	0.001*
T lymphocytes CD3+CD4+ [%] & T lymphocytes ratio CD3+CD4+/T CD3+CD8+	0.762	4.561	0.000*
Stage (TNM) [I-IV] & TLR-9 serum concentration [ng/mL]	0.788	4.957	0.000*
T lymphocytes CD4+TLR-9+ [%] & T lymphocytes CD8+TLR-9+ [%]	0.858	6.465	0.000*
NK cells CD3-CD16+CD56+ [%] & NKT-like cells CD3+CD16+CD56+ [%]	0.963	13.886	0.000*

Supplementary Materials Table S6: Sperman's rank correlation analysis in GC-G1 patients.

Parameters	R	t(N-2)	p
T lymphocytes ratio CD3+CD4+/T CD3+CD8+ & T lymphocytes CD3+CD8+ [%]	-0.964	-10.200	0.000*
T lymphocytes ratio CD3+CD4+/T CD3+CD8+ & NKT-like cells CD3+CD16+CD56+ [%]	-0.879	-5.209	0.001*
NKT-like cells CD3+CD16+CD56+ [%] & Survival time from diagnosis [months]	-0.830	-4.214	0.003*
T lymphocytes ratio CD3+CD4+/T CD3+CD8+ & NK cells CD3-CD16+CD56+ [%]	-0.806	-3.852	0.005*
T lymphocytes CD3+CD8+ [%] & T lymphocytes CD3+CD4+ [%]	-0.794	-3.693	0.006*
White blood cells [10^3/mm^3] & NKT-like cells CD3+CD16+CD56+ [%]	-0.733	-3.051	0.016*
NK cells CD3-CD16+CD56+ [%] & T lymphocytes CD3+CD4+ [%]	-0.673	-2.572	0.033*
B lymphocytes CD19+TLR-9+ [%] & Lymphocytes [10^3/mm^3]	-0.661	-2.489	0.038*
NK cells CD3-CD16+CD56+ [%] & T lymphocytes CD8+TLR-9+ [%]	-0.648	-2.410	0.043*
T lymphocytes CD3+CD8+ [%] & Monocytes [10^3/mm^3]	0.638	2.345	0.047*
Survival time from diagnosis [months] & T lymphocytes ratio CD3+CD4+/T CD3+CD8+	0.697	2.749	0.025*
NK cells CD3-CD16+CD56+ [%] & T lymphocytes CD3+CD8+ [%]	0.745	3.163	0.013*

NK cells CD3-CD16+CD56+ [%] & NKT-like cells CD3+CD16+CD56+ [%]	0.879	5.209	0.001*
NKT-like cells CD3+CD16+CD56+ [%] & T lymphocytes CD3+CD8+ [%]	0.879	5.209	0.001*
T lymphocytes CD3+CD8+ [%] & NKT-like cells CD3+CD16+CD56+ [%]	0.879	5.209	0.001*
Survival time from diagnosis [months] & White blood cells [10^3/mm^3]	0.891	5.548	0.001*

Supplementary Materials Table S7: Sperman's rank correlation analysis in GC-G2 patients.

Parameters	R	t(N-2)	p
White blood cells [10^3/mm^3] & NKT-like cells CD3+CD16+CD56+ [%]	-0.995	-31.508	0.000*
White blood cells [10^3/mm^3] & NK cells CD3-CD16+CD56+ [%]	-0.989	-22.187	0.000*
Survival time from diagnosis [months] & TLR-9 serum concentration [ng/mL]	-0.941	-9.210	0.000*
T lymphocytes CD3+CD8+ [%] & T lymphocytes ratio CD3+CD4+/T CD3+CD8+	-0.912	-7.378	0.000*
Survival time from diagnosis [months] & DNA EBV copy number [copies/1 ug DNA]	-0.897	-6.732	0.000*
Survival time from diagnosis [months] & T lymphocytes CD4+TLR-9+ [%]	-0.856	-5.481	0.000*
Stage (TNM) [I-IV] & Survival time from diagnosis [months]	-0.762	-3.899	0.002*
Survival time from diagnosis [months] & T lymphocytes CD8+TLR-9+ [%]	-0.754	-3.804	0.003*
Survival time from diagnosis [months] & B lymphocytes CD19+TLR-9+ [%]	-0.740	-3.649	0.004*
Lymphocytes [10^3/mm^3] & NK cells CD3-CD16+CD56+ [%]	-0.698	-3.231	0.008*
Lymphocytes [10^3/mm^3] & NKT-like cells CD3+CD16+CD56+ [%]	-0.692	-3.182	0.009*
NK cells CD3-CD16+CD56+ [%] & T lymphocytes ratio CD3+CD4+/T CD3+CD8+	-0.637	-2.743	0.019*
White blood cells [10^3/mm^3] & T lymphocytes CD3+CD8+ [%]	-0.582	-2.376	0.037*
T lymphocytes CD3+CD8+ [%] & White blood cells [10^3/mm^3]	-0.582	-2.376	0.037*
T lymphocytes CD3+CD4+ [%] & T lymphocytes CD4+TLR-9+ [%]	-0.555	-2.212	0.049*
T lymphocytes CD3+CD8+ [%] & NKT-like cells CD3+CD16+CD56+ [%]	0.588	2.410	0.035*

**T lymphocytes ratio CD3+CD4+/T CD3+CD8+ & White blood cells [10^3/mm^3]**	0.599	2.480	0.031*
**B lymphocytes CD19+TLR-9+ [%] & Monocytes [10^3/mm^3]**	0.605	2.522	0.028*
**T lymphocytes CD3+CD8+ [%] & T lymphocytes CD3+ [%]**	0.626	2.665	0.022*
**T lymphocytes CD3+CD8+ [%] & NK cells CD3-CD16+CD56+ [%]**	0.637	2.743	0.019*
**B lymphocytes CD19+TLR-9+ [%] & T lymphocytes CD4+TLR-9+ [%]**	0.648	2.824	0.017*
**B lymphocytes CD19+TLR-9+ [%] & Stage (TNM) [I-IV]**	0.676	3.044	0.011*
**Stage (TNM) [I-IV] & TLR-9 serum concentration [ng/mL]**	0.718	3.425	0.006*
**TLR-9 serum concentration [ng/mL] & Stage (TNM) [I-IV]**	0.718	3.425	0.006*
**TLR-9 serum concentration [ng/mL] & B lymphocytes CD19+TLR-9+ [%]**	0.731	3.551	0.005*
**Lymphocytes [10^3/mm^3] & White blood cells [10^3/mm^3]**	0.747	3.729	0.003*
**DNA EBV copy number [copies/1 ug DNA] & Stage (TNM) [I-IV]**	0.778	4.105	0.002*
**T lymphocytes CD8+TLR-9+ [%] & B lymphocytes CD19+TLR-9+ [%]**	0.786	4.213	0.001*
**T lymphocytes CD4+TLR-9+ [%] & TLR-9 serum concentration [ng/mL]**	0.797	4.372	0.001*
**DNA EBV copy number [copies/1 ug DNA] & T lymphocytes CD4+TLR-9+ [%]**	0.800	4.429	0.001*
**Stage (TNM) [I-IV] & T lymphocytes CD4+TLR-9+ [%]**	0.803	4.467	0.001*
**T lymphocytes CD8+TLR-9+ [%] & TLR-9 serum concentration [ng/mL]**	0.813	4.634	0.001*
**DNA EBV copy number [copies/1 ug DNA] & T lymphocytes CD8+TLR-9+ [%]**	0.824	4.815	0.001*
**T lymphocytes CD4+TLR-9+ [%] & T lymphocytes CD8+TLR-9+ [%]**	0.830	4.929	0.000*
**DNA EBV copy number [copies/1 ug DNA] & B lymphocytes CD19+TLR-9+ [%]**	0.899	6.796	0.000*
**B lymphocytes CD19+TLR-9+ [%] & DNA EBV copy number [copies/1 ug DNA]**	0.899	6.796	0.000*
**NK cells CD3-CD16+CD56+ [%] & NKT-like cells CD3+CD16+CD56+ [%]**	0.995	31.508	0.000*

Supplementary Materials Table S8: Sperman's rank correlation analysis in GC-G3 patients.

**Parameters**	**R**	**t(N-2)**	**p**
**White blood cells [10^3/mm^3] & NK cells CD3-CD16+CD56+ [%]**	-0.998	-55.216	0.000*

White blood cells [10^3/mm^3] & NKT-like cells CD3+CD16+CD56+ [%]	-0.992	-30.492	0.000*
Monocytes [10^3/mm^3] & NK cells CD3-CD16+CD56+ [%]	-0.991	-29.361	0.000*
Monocytes [10^3/mm^3] & NKT-like cells CD3+CD16+CD56+ [%]	-0.988	-24.488	0.000*
Lymphocytes [10^3/mm^3] & NK cells CD3-CD16+CD56+ [%]	-0.985	-21.865	0.000*
Lymphocytes [10^3/mm^3] & NKT-like cells CD3+CD16+CD56+ [%]	-0.981	-19.574	0.000*
White blood cells [10^3/mm^3] & T lymphocytes CD3+CD8+ [%]	-0.941	-10.787	0.000*
Monocytes [10^3/mm^3] & T lymphocytes CD3+CD8+ [%]	-0.937	-10.427	0.000*
Lymphocytes [10^3/mm^3] & T lymphocytes CD3+CD8+ [%]	-0.931	-9.856	0.000*
T lymphocytes CD3+CD8+ [%] & T lymphocytes ratio CD3+CD4+/T CD3+CD8+	-0.909	-8.463	0.000*
Survival time from diagnosis [months] & T lymphocytes CD8+TLR-9+ [%]	-0.886	-7.412	0.000*
Survival time from diagnosis [months] & DNA EBV copy number [copies/1 ug DNA]	-0.874	-6.956	0.000*
NKT-like cells CD3+CD16+CD56+ [%] & T lymphocytes ratio CD3+CD4+/T CD3+CD8+	-0.866	-6.699	0.000*
NK cells CD3-CD16+CD56+ [%] & T lymphocytes ratio CD3+CD4+/T CD3+CD8+	-0.858	-6.465	0.000*
Survival time from diagnosis [months] & TLR-9 serum concentration [ng/mL]	-0.781	-4.837	0.000*
Survival time from diagnosis [months] & B lymphocytes CD19+TLR-9+ [%]	-0.739	-4.246	0.001*
Survival time from diagnosis [months] & T lymphocytes CD4+TLR-9+ [%]	-0.655	-3.359	0.004*
T lymphocytes CD3+CD4+ [%] & T lymphocytes CD3+CD8+ [%]	-0.581	-2.764	0.014*
NKT-like cells CD3+CD16+CD56+ [%] & T lymphocytes CD3+CD4+ [%]	-0.554	-2.579	0.021*
NK cells CD3-CD16+CD56+ [%] & T lymphocytes CD3+CD4+ [%]	-0.534	-2.448	0.027*
Stage (TNM) [I-IV] & Survival time from diagnosis [months]	-0.524	-2.384	0.031*
Lymphocytes [10^3/mm^3] & T lymphocytes CD4+TLR-9+ [%]	0.495	2.209	0.043*
White blood cells [10^3/mm^3] & T lymphocytes CD3+CD4+ [%]	0.515	2.325	0.035*
T lymphocytes CD3+CD4+ [%] & White blood cells [10^3/mm^3]	0.515	2.325	0.035*

Monocytes [10^3/mm^3] & T lymphocytes CD3+CD4+ [%]	0.520	2.359	0.032*
Stage (TNM) [I-IV] & T lymphocytes CD4+TLR-9+ [%]	0.526	2.394	0.030*
Stage (TNM) [I-IV] & T lymphocytes CD8+TLR-9+ [%]	0.560	2.619	0.019*
T lymphocytes CD4+TLR-9+ [%] & B lymphocytes CD19+TLR-9+ [%]	0.596	2.872	0.012*
Stage (TNM) [I-IV] & TLR-9 serum concentration [ng/mL]	0.601	2.914	0.011*
Stage (TNM) [I-IV] & DNA EBV copy number [copies/1 ug DNA]	0.602	2.920	0.011*
T lymphocytes CD4+TLR-9+ [%] & TLR-9 serum concentration [ng/mL]	0.637	3.203	0.006*
T lymphocytes CD4+TLR-9+ [%] & T lymphocytes CD8+TLR-9+ [%]	0.762	4.561	0.000*
DNA EBV copy number [copies/1 ug DNA] & T lymphocytes CD4+TLR-9+ [%]	0.774	4.738	0.000*
T lymphocytes CD3+CD4+ [%] & T lymphocytes ratio CD3+CD4+/T CD3+CD8+	0.809	5.327	0.000*
B lymphocytes CD19+TLR-9+ [%] & TLR-9 serum concentration [ng/mL]	0.816	5.471	0.000*
Lymphocytes [10^3/mm^3] & T lymphocytes ratio CD3+CD4+/T CD3+CD8+	0.830	5.767	0.000*
Monocytes [10^3/mm^3] & T lymphocytes ratio CD3+CD4+/T CD3+CD8+	0.833	5.834	0.000*
T lymphocytes CD8+TLR-9+ [%] & B lymphocytes CD19+TLR-9+ [%]	0.846	6.135	0.000*
White blood cells [10^3/mm^3] & T lymphocytes ratio CD3+CD4+/T CD3+CD8+	0.853	6.328	0.000*
DNA EBV copy number [copies/1 ug DNA] & B lymphocytes CD19+TLR-9+ [%]	0.854	6.348	0.000*
T lymphocytes CD8+TLR-9+ [%] & TLR-9 serum concentration [ng/mL]	0.900	7.974	0.000*
NK cells CD3-CD16+CD56+ [%] & T lymphocytes CD3+CD8+ [%]	0.946	11.311	0.000*
NKT-like cells CD3+CD16+CD56+ [%] & T lymphocytes CD3+CD8+ [%]	0.948	11.522	0.000*
DNA EBV copy number [copies/1 ug DNA] & TLR-9 serum concentration [ng/mL]	0.955	12.531	0.000*
DNA EBV copy number [copies/1 ug DNA] & T lymphocytes CD8+TLR-9+ [%]	0.968	14.895	0.000*
Monocytes [10^3/mm^3] & Lymphocytes [10^3/mm^3]	0.977	17.861	0.000*

**White blood cells [10^3/mm^3] & Monocytes [10^3/mm^3]**	0.987	23.334	0.000*
**White blood cells [10^3/mm^3] & Lymphocytes [10^3/mm^3]**	0.992	30.492	0.000*
**NK cells CD3-CD16+CD56+ [%] & NKT-like cells CD3+CD16+CD56+ [%]**	0.997	49.350	0.000*

Supplementary Materials Table S9: Sperman's rank correlation analysis in GC-TNM I patients.

**Parameters**	**R**	**t(N-2)**	**p**
**T lymphocytes ratio CD3+CD4+/T CD3+CD8+ & T lymphocytes CD3+CD8+ [%]**	-0.964	-8.141	0.000*
**Survival time from diagnosis [months] & NKT-like cells CD3+CD16+CD56+ [%]**	-0.929	-5.594	0.003*
**NKT-like cells CD3+CD16+CD56+ [%] & Survival time from diagnosis [months]**	-0.929	-5.594	0.003*
**T lymphocytes CD3+CD8+ [%] & T lymphocytes CD3+CD4+ [%]**	-0.929	-5.594	0.003*
**NKT-like cells CD3+CD16+CD56+ [%] & T lymphocytes ratio CD3+CD4+/T CD3+CD8+**	-0.893	-4.433	0.007*
**T lymphocytes ratio CD3+CD4+/T CD3+CD8+ & NKT-like cells CD3+CD16+CD56+ [%]**	-0.893	-4.433	0.007*
**NKT-like cells CD3+CD16+CD56+ [%] & T lymphocytes CD3+CD4+ [%]**	-0.857	-3.721	0.014*
**T lymphocytes CD3+CD4+ [%] & NKT-like cells CD3+CD16+CD56+ [%]**	-0.857	-3.721	0.014*
**White blood cells [10^3/mm^3] & NKT-like cells CD3+CD16+CD56+ [%]**	-0.786	-2.840	0.036*
**NK cells CD3-CD16+CD56+ [%] & White blood cells [10^3/mm^3]**	-0.786	-2.840	0.036*
**T lymphocytes CD3+CD8+ [%] & T lymphocytes CD8+TLR-9+ [%]**	-0.786	-2.840	0.036*
**NK cells CD3-CD16+CD56+ [%] & T lymphocytes CD3+CD8+ [%]**	0.786	2.840	0.036*
**T lymphocytes CD3+CD8+ [%] & NK cells CD3-CD16+CD56+ [%]**	0.786	2.840	0.036*

**T lymphocytes ratio CD3+CD4+/T CD3+CD8+ & T lymphocytes CD8+TLR-9+ [%]**	0.821	3.221	0.023*
**Survival time from diagnosis [months] & White blood cells [10^3/mm^3]**	0.857	3.721	0.014*
**T lymphocytes CD3+CD4+ [%] & T lymphocytes CD8+TLR-9+ [%]**	0.929	5.594	0.003*
**T lymphocytes CD3+CD4+ [%] & T lymphocytes ratio CD3+CD4+/T CD3+CD8+**	0.964	8.141	0.000*

Supplementary Materials Table S10: Sperman's rank correlation analysis in GC-TNM II patients.

**Parameters**	**R**	**t(N-2)**	**p**
**White blood cells [10^3/mm^3] & NKT-like cells CD3+CD16+CD56+ [%]**	-0.979	-15.194	0.000*
**NKT-like cells CD3+CD16+CD56+ [%] & T lymphocytes ratio CD3+CD4+/T CD3+CD8+**	-0.965	-11.642	0.000*
**T lymphocytes ratio CD3+CD4+/T CD3+CD8+ & NKT-like cells CD3+CD16+CD56+ [%]**	-0.965	-11.642	0.000*
**White blood cells [10^3/mm^3] & NK cells CD3-CD16+CD56+ [%]**	-0.888	-6.110	0.000*
**NK cells CD3-CD16+CD56+ [%] & T lymphocytes ratio CD3+CD4+/T CD3+CD8+**	-0.888	-6.110	0.000*
**White blood cells [10^3/mm^3] & T lymphocytes CD3+CD8+ [%]**	-0.874	-5.691	0.000*
**NKT-like cells CD3+CD16+CD56+ [%] & T lymphocytes CD3+CD4+ [%]**	-0.825	-4.619	0.001*
**Survival time from diagnosis [months] & White blood cells [10^3/mm^3]**	-0.783	-3.984	0.003*
**T lymphocytes CD3+CD4+ [%] & T lymphocytes CD3+CD8+ [%]**	-0.769	-3.807	0.003*
**NK cells CD3-CD16+CD56+ [%] & T lymphocytes CD3+CD4+ [%]**	-0.748	-3.567	0.005*
**Survival time from diagnosis [months] & T lymphocytes ratio CD3+CD4+/T CD3+CD8+**	-0.720	-3.284	0.008*
**Age & TLR-9 serum concentration [ng/mL]**	-0.620	-2.499	0.032*

**Survival time from diagnosis [months] & T lymphocytes CD3+CD8+ [%]**	0.636	2.609	0.026*
**Monocytes [10^3/mm^3] & Lymphocytes [10^3/mm^3]**	0.755	3.644	0.005*
**Survival time from diagnosis [months] & NKT-like cells CD3+CD16+CD56+ [%]**	0.762	3.724	0.004*
**NKT-like cells CD3+CD16+CD56+ [%] & Survival time from diagnosis [months]**	0.762	3.724	0.004*
**T lymphocytes CD3+CD4+ [%] & White blood cells [10^3/mm^3]**	0.790	4.078	0.002*
**NK cells CD3-CD16+CD56+ [%] & Survival time from diagnosis [months]**	0.797	4.176	0.002*
**T lymphocytes CD3+CD8+ [%] & NK cells CD3-CD16+CD56+ [%]**	0.853	5.172	0.000*
**T lymphocytes ratio CD3+CD4+/T CD3+CD8+ & T lymphocytes CD3+CD4+ [%]**	0.867	5.505	0.000*
**T lymphocytes CD3+CD8+ [%] & NKT-like cells CD3+CD16+CD56+ [%]**	0.916	7.224	0.000*
**NKT-like cells CD3+CD16+CD56+ [%] & NK cells CD3-CD16+CD56+ [%]**	0.923	7.589	0.000*
**T lymphocytes ratio CD3+CD4+/T CD3+CD8+ & White blood cells [10^3/mm^3]**	0.944	9.052	0.000*

Supplementary Materials Table S11: Sperman's rank correlation analysis in GC-TNM III patients.

**Parameters**	**R**	**t(N-2)**	**p**
**White blood cells [10^3/mm^3] & NK cells CD3-CD16+CD56+ [%]**	-0.978	-15.557	0.000*
**White blood cells [10^3/mm^3] & NKT-like cells CD3+CD16+CD56+ [%]**	-0.967	-12.595	0.000*
**T lymphocytes CD3+CD8+ [%] & T lymphocytes ratio CD3+CD4+/T CD3+CD8+**	-0.945	-9.588	0.000*
**Survival time from diagnosis [months] & TLR-9 serum concentration [ng/mL]**	-0.827	-4.873	0.000*
**NK cells CD3-CD16+CD56+ [%] & T lymphocytes ratio CD3+CD4+/T CD3+CD8+**	-0.824	-4.827	0.001*
**Survival time from diagnosis [months] & DNA EBV copy number [copies/1 ug DNA]**	-0.811	-4.600	0.001*
**Lymphocytes [10^3/mm^3] & NK cells CD3-CD16+CD56+ [%]**	-0.791	-4.291	0.001*

**White blood cells [10^3/mm^3] & T lymphocytes CD3+CD8+ [%]**	-0.764	-3.924	0.002*
**Lymphocytes [10^3/mm^3] & NKT-like cells CD3+CD16+CD56+ [%]**	-0.742	-3.668	0.004*
**Survival time from diagnosis [months] & T lymphocytes CD4+TLR-9+ [%]**	-0.727	-3.510	0.005*
**NKT-like cells CD3+CD16+CD56+ [%] & T lymphocytes ratio CD3+CD4+/T CD3+CD8+**	-0.709	-3.332	0.007*
**Lymphocytes [10^3/mm^3] & T lymphocytes CD3+CD8+ [%]**	-0.665	-2.952	0.013*
**Monocytes [10^3/mm^3] & NKT-like cells CD3+CD16+CD56+ [%]**	-0.641	-2.770	0.018*
**Monocytes [10^3/mm^3] & NK cells CD3-CD16+CD56+ [%]**	-0.608	-2.540	0.027*
**T lymphocytes CD3+CD4+ [%] & T lymphocytes CD8+TLR-9+ [%]**	-0.588	-2.410	0.035*
**Survival time from diagnosis [months] & T lymphocytes CD8+TLR-9+ [%]**	-0.555	-2.212	0.049*
**T lymphocytes CD3+CD4+ [%] & T lymphocytes ratio CD3+CD4+/T CD3+CD8+**	0.555	2.212	0.049*
**T lymphocytes CD4+TLR-9+ [%] & T lymphocytes CD8+TLR-9+ [%]**	0.637	2.743	0.019*
**Lymphocytes [10^3/mm^3] & T lymphocytes ratio CD3+CD4+/T CD3+CD8+**	0.643	2.783	0.018*
**B lymphocytes CD19+TLR-9+ [%] & TLR-9 serum concentration [ng/mL]**	0.643	2.783	0.018*
**DNA EBV copy number [copies/1 ug DNA] & T lymphocytes CD8+TLR-9+ [%]**	0.646	2.810	0.017*
**NKT-like cells CD3+CD16+CD56+ [%] & T lymphocytes CD3+CD8+ [%]**	0.676	3.041	0.011*
**T lymphocytes CD8+TLR-9+ [%] & TLR-9 serum concentration [ng/mL]**	0.720	3.439	0.006*
**DNA EBV copy number [copies/1 ug DNA] & B lymphocytes CD19+TLR-9+ [%]**	0.751	3.773	0.003*
**White blood cells [10^3/mm^3] & T lymphocytes ratio CD3+CD4+/T CD3+CD8+**	0.775	4.064	0.002*
**DNA EBV copy number [copies/1 ug DNA] & T lymphocytes CD4+TLR-9+ [%]**	0.795	4.347	0.001*
**T lymphocytes CD4+TLR-9+ [%] & TLR-9 serum concentration [ng/mL]**	0.808	4.543	0.001*
**NK cells CD3-CD16+CD56+ [%] & T lymphocytes CD3+CD8+ [%]**	0.819	4.728	0.001*
**White blood cells [10^3/mm^3] & Lymphocytes [10^3/mm^3]**	0.835	5.036	0.000*
**DNA EBV copy number [copies/1 ug DNA] & TLR-9 serum concentration [ng/mL]**	0.949	9.995	0.000*
**NK cells CD3-CD16+CD56+ [%] & NKT-like cells CD3+CD16+CD56+ [%]**	0.967	12.595	0.000*

Supplementary Materials Table S12: Sperman's rank correlation analysis in GC-TNM IV patients.

**Parameters**	**R**	**t(N-2)**	**p**

DNA EBV copy number [copies/1 ug DNA] & NKT-like cells CD3+CD16+CD56+ [%]	-0.994	-22.316	0.000*
Monocytes [10^3/mm^3] & NK cells CD3-CD16+CD56+ [%]	-0.994	-22.316	0.000*
Lymphocytes [10^3/mm^3] & NK cells CD3-CD16+CD56+ [%]	-0.994	-22.316	0.000*
NKT-like cells CD3+CD16+CD56+ [%] & DNA EBV copy number [copies/1 ug DNA]	-0.994	-22.316	0.000*
NKT-like cells CD3+CD16+CD56+ [%] & White blood cells [10^3/mm^3]	-0.994	-22.316	0.000*
Lymphocytes [10^3/mm^3] & NKT-like cells CD3+CD16+CD56+ [%]	-0.982	-12.709	0.000*
NKT-like cells CD3+CD16+CD56+ [%] & Monocytes [10^3/mm^3]	-0.982	-12.709	0.000*
NK cells CD3-CD16+CD56+ [%] & T lymphocytes CD8+TLR-9+ [%]	-0.976	-11.024	0.000*
NKT-like cells CD3+CD16+CD56+ [%] & Neutrophils [10^3/mm^3]	-0.970	-9.787	0.000*
T lymphocytes CD8+TLR-9+ [%] & NKT-like cells CD3+CD16+CD56+ [%]	-0.970	-9.787	0.000*
T lymphocytes CD4+TLR-9+ [%] & NK cells CD3-CD16+CD56+ [%]	-0.929	-6.128	0.001*
T lymphocytes CD4+TLR-9+ [%] & NKT-like cells CD3+CD16+CD56+ [%]	-0.898	-5.006	0.002*
NK cells CD3-CD16+CD56+ [%] & T lymphocytes CD3+ [%]	-0.857	-4.076	0.007*
NKT-like cells CD3+CD16+CD56+ [%] & T lymphocytes CD3+ [%]	-0.850	-3.958	0.007*
NKT-like cells CD3+CD16+CD56+ [%] & TLR-9 serum concentration [ng/mL]	-0.826	-3.594	0.011*
TLR-9 serum concentration [ng/mL] & NKT-like cells CD3+CD16+CD56+ [%]	-0.826	-3.594	0.011*
B lymphocytes CD19+TLR-9+ [%] & NK cells CD3-CD16+CD56+ [%]	-0.810	-3.378	0.015*
T lymphocytes CD3+CD8+ [%] & T lymphocytes ratio CD3+CD4+/T CD3+CD8+	-0.786	-3.111	0.021*
TLR-9 serum concentration [ng/mL] & NK cells CD3-CD16+CD56+ [%]	-0.786	-3.111	0.021*
T lymphocytes CD4+TLR-9+ [%] & Survival time from diagnosis [months]	-0.771	-2.967	0.025*
Survival time from diagnosis [months] & T lymphocytes CD3+ [%]	-0.711	-2.476	0.048*
Monocytes [10^3/mm^3] & TLR-9 serum concentration [ng/mL]	0.731	2.621	0.040*
TLR-9 serum concentration [ng/mL] & Monocytes [10^3/mm^3]	0.731	2.621	0.040*
T lymphocytes CD3+CD4+ [%] & TLR-9 serum concentration [ng/mL]	0.738	2.680	0.037*
T lymphocytes CD8+TLR-9+ [%] & TLR-9 serum concentration [ng/mL]	0.762	2.881	0.028*
B lymphocytes CD19+TLR-9+ [%] & TLR-9 serum concentration [ng/mL]	0.762	2.881	0.028*
TLR-9 serum concentration [ng/mL] & B lymphocytes CD19+TLR-9+ [%]	0.762	2.881	0.028*
White blood cells [10^3/mm^3] & TLR-9 serum concentration [ng/mL]	0.786	3.111	0.021*

TLR-9 serum concentration [ng/mL] & DNA EBV copy number [copies/1 ug DNA]	0.786	3.111	0.021*
Monocytes [10^3/mm^3] & B lymphocytes CD19+TLR-9+ [%]	0.790	3.161	0.020*
B lymphocytes CD19+TLR-9+ [%] & Monocytes [10^3/mm^3]	0.790	3.161	0.020*
DNA EBV copy number [copies/1 ug DNA] & B lymphocytes CD19+TLR-9+ [%]	0.810	3.378	0.015*
T lymphocytes ratio CD3+CD4+/T CD3+CD8+ & TLR-9 serum concentration [ng/mL]	0.810	3.378	0.015*
B lymphocytes CD19+TLR-9+ [%] & White blood cells [10^3/mm^3]	0.810	3.378	0.015*
T lymphocytes CD3+ [%] & T lymphocytes CD8+TLR-9+ [%]	0.833	3.693	0.010*
T lymphocytes ratio CD3+CD4+/T CD3+CD8+ & T lymphocytes CD3+CD4+ [%]	0.833	3.693	0.010*
DNA EBV copy number [copies/1 ug DNA] & T lymphocytes CD3+ [%]	0.857	4.076	0.007*
White blood cells [10^3/mm^3] & T lymphocytes CD3+ [%]	0.857	4.076	0.007*
T lymphocytes CD3+ [%] & White blood cells [10^3/mm^3]	0.857	4.076	0.007*
T lymphocytes CD4+TLR-9+ [%] & T lymphocytes CD3+ [%]	0.881	4.560	0.004*
Lymphocytes [10^3/mm^3] & T lymphocytes CD3+ [%]	0.886	4.686	0.003*
T lymphocytes CD3+ [%] & Lymphocytes [10^3/mm^3]	0.886	4.686	0.003*
T lymphocytes CD4+TLR-9+ [%] & T lymphocytes CD8+TLR-9+ [%]	0.905	5.203	0.002*
DNA EBV copy number [copies/1 ug DNA] & T lymphocytes CD4+TLR-9+ [%]	0.929	6.128	0.001*
T lymphocytes CD4+TLR-9+ [%] & DNA EBV copy number [copies/1 ug DNA]	0.929	6.128	0.001*
T lymphocytes CD8+TLR-9+ [%] & Neutrophils [10^3/mm^3]	0.952	7.651	0.000*
T lymphocytes CD4+TLR-9+ [%] & Monocytes [10^3/mm^3]	0.958	8.193	0.000*
Monocytes [10^3/mm^3] & T lymphocytes CD8+TLR-9+ [%]	0.970	9.787	0.000*
T lymphocytes CD8+TLR-9+ [%] & Monocytes [10^3/mm^3]	0.970	9.787	0.000*
T lymphocytes CD8+TLR-9+ [%] & DNA EBV copy number [copies/1 ug DNA]	0.976	11.024	0.000*
DNA EBV copy number [copies/1 ug DNA] & Monocytes [10^3/mm^3]	0.994	22.316	0.000*
White blood cells [10^3/mm^3] & Monocytes [10^3/mm^3]	0.994	22.316	0.000*
Monocytes [10^3/mm^3] & DNA EBV copy number [copies/1 ug DNA]	0.994	22.316	0.000*
Lymphocytes [10^3/mm^3] & DNA EBV copy number [copies/1 ug DNA]	0.994	22.316	0.000*
NK cells CD3-CD16+CD56+ [%] & NKT-like cells CD3+CD16+CD56+ [%]	0.994	22.316	0.000*
